# Glutathione S-Transferase of Brown Planthoppers (*Nilaparvata lugens*) Is Essential for Their Adaptation to Gramine-Containing Host Plants

**DOI:** 10.1371/journal.pone.0064026

**Published:** 2013-05-20

**Authors:** Xiao-Qin Sun, Mao-Xin Zhang, Jing-Ya Yu, Yu Jin, Bing Ling, Jin-Ping Du, Gui-Hua Li, Qing-Ming Qin, Qing-Nian Cai

**Affiliations:** 1 College of Agronomy and Biotechnology, China Agricultural University, Beijing, China; 2 College of Natural Resources and Environment, South China Agricultural University, Guangzhou, China; 3 College of Plant Science and Technology, Huazhong Agricultural University, Wuhan, China; 4 College of Plant Sciences, Jilin University, Changchun, China; 5 Key Laboratory of Zoonosis Research, Ministry of Education, Institute of Zoonosis, Jilin University, Changchun, China; Natural Resources Canada, Canada

## Abstract

Plants have evolved complex processes to ward off attacks by insects. In parallel, insects have evolved mechanisms to thwart these plant defenses. To gain insight into mechanisms that mediate this arms race between plants and herbivorous insects, we investigated the interactions between gramine, a toxin synthesized by plants of the family *Gramineae*, and glutathione S transferase (GST), an enzyme found in insects that is known to detoxify xenobiotics. Here, we demonstrate that rice (*Oryza sativa*), a hydrophytic plant, also produces gramine and that rice resistance to brown planthoppers (*Nilaparvata lugens,* BPHs) is highly associated with *in planta* gramine content. We also show that gramine is a toxicant that causes BPH mortality *in vivo* and that knockdown of BPH GST gene *nlgst1-1* results in increased sensitivity to diets containing gramine. These results suggest that the knockdown of key detoxification genes in sap-sucking insects may provide an avenue for increasing their sensitivity to natural plant-associated defense mechanisms.

## Introduction

The brown planthopper (BPH), *Nilaparvata lugens* Stål. (Homoptera: Delphacidae), one of the most devastating pests in many rice (*Oryza sativa*) growing areas in Asia, causes physiological damage to rice crops by direct feeding, and by acting as vectors for the transmission of viruses that cause ragged stunt diseases on host plants [1]. Insecticides have proven efficacious in controlling BPH population [2]. However, widespread insecticide use potentially threatens the environment and food safety in agroecosystems. As an alternative strategy, some BPH resistant rice cultivars have been developed to protect rice plants from BPH attacks [3]–[6]. Although this strategy has proven successful in controlling BPH population, the ability of this pest to overcome the natural defenses of rice plants poses challenges to rice production [7]. To develop a better strategy for controlling BPH populations, some efforts have been made to explore the effect of rice secondary metabolites on the control of BPH population [8]–[11]. However, the involvement of secondary metabolites (volatiles and secondary chemicals) derived from rice plants in defense against BPHs, and the mechanisms mediating their activities, remains obscure [12].

The production of secondary metabolites including alkaloids, phenolics and peptides by plants has long been regarded as an important strategy for plant defense against herbivores. Plant alkaloids are known as toxicants to many herbivorous insects. They are also regarded as a resource for the discovery of novel biological insecticides [13]. Gramine is a simple indole alkaloid found only in some terrestrial gramineous crops (*Triticeae* crops) [14], and has been reported to be a deterrent and/or toxicant to some herbivorous insects such as aphids (*Rhopalosiphum padi* and *Sitobion avenae*) and cotton bollworm (*Helicoverpa armigera*) [15]–[17]. However, little is known whether gramine is synthesized by hydrophytic plants such as rice, or whether this compound has the ability to confer resistance to attacks by BPHs.

On the other hand, herbivorous insects have developed a set of detoxification enzymes that help address the challenges posed by plants or synthetic xenobiotics. These enzymes include cytochrome P450s, glutathione S-transferases (GSTs) and carboxylesterases/cholinesterases that help to protect insects from these agents [18]–[20]. In insects, GSTs play an essential role in mediating the detoxification of xenobiotics including many insecticides such as organophosphate, organochlorines and pyrethroids [21]–[23] and host plant secondary metabolites, including indole-3-acetonitrile and xanthotoxin [24], [25]. Recently, nine GST EST (expressed sequence tags) sequences were identified in BPHs [26]. Among all those genes, *nlgst1-1* [18] (GenBank accession no: AF448500), a class-Delta GST gene that confers detoxification functions [21], [27], was the only one that proved to be involved with insecticide resistance [21]. Moreover, compared with other tissues/organs, the BPH GST gene *nlgst1-1* was highly expressed in BPH intestine [28], suggesting that plant metabolites ingested by the pests may be detoxified in this organ. However, to date, little is known about the metabolic role of BPH GSTs in the detoxification of plant secondary metabolites.

In the present study, we provide evidence to demonstrate that (1) rice plants contain gramine; (2) the gramine content in rice plants is associated with their resistance to BPHs; (3) gramine is toxic to BPHs, and (4) the BPH GST gene *nlgst1-1* is essential for gramine detoxification and adaptation to gramine-containing host plants. Our findings provide new insight into molecular mechanisms mediating BPH-rice interaction, and may result in the development of more effective and practical strategies for controlling sap-sucking pests such as BPHs.

## Results

### Gramine Content in Various Rice Cultivars

**Figure 1:**
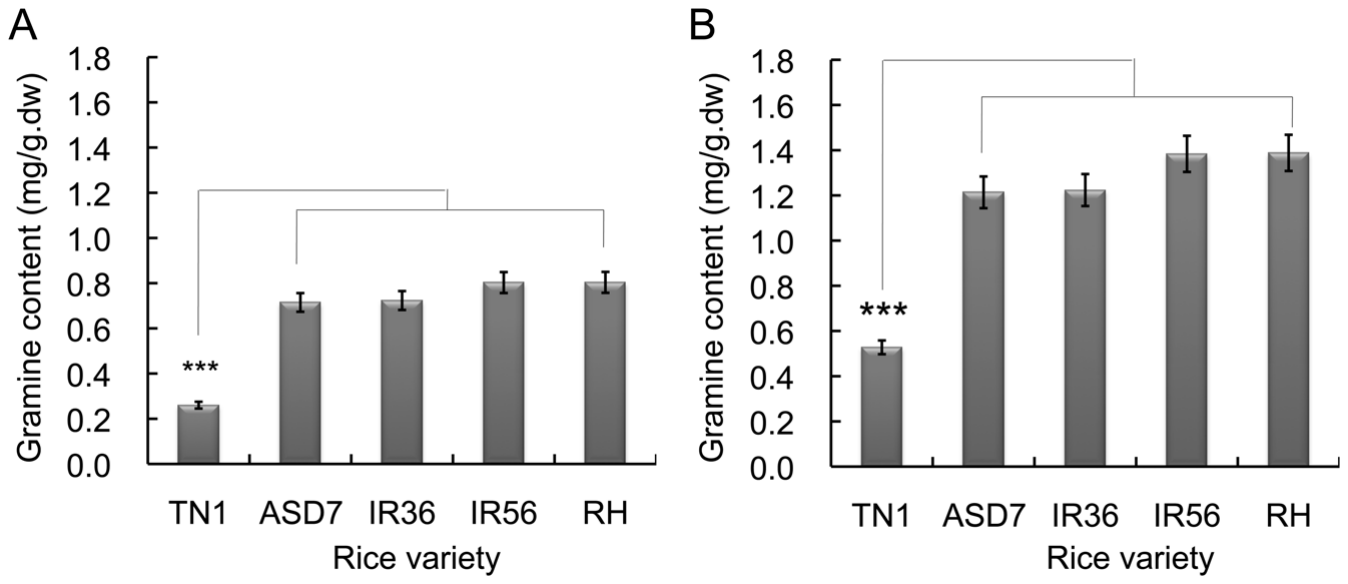
Gramine content in various rice cultivars. Gramine content was extracted from brown planthopper (BPH)-resistant and BPH-susceptible rice varieties at the seedling (**A**) and tillering (**B**) stages. The gramine content was determined by HLPC approach as described in *Materials and Methods*. Data represent the means ± standard deviation (SD) from three independent experiments with triplicate samples (each cultivar containing 10 seedling or tillering rice plants) examined. ***indicates significance at *P*<0.001. dw: dry weight. TN1: rice cultivar Taichung Natuva 1 (BPH susceptible); ADS7, IR36, IR56 and RH (Rathu Heenati): BPH resistant rice varieties.

To investigate whether gramine exists in rice crops, we analyzed gramine levels in rice plants with BPH-resistant and BPH-susceptible cultivars at seedling and tillering stages using previously described HPLC analysis method [29]. Gramine content in BPH-resistant rice cultivars was significantly higher than that in the BPH-susceptible cultivar at both seedling and tillering stages (Fig. 1). Of the tested rice cultivars, TN1, a BPH-susceptible rice cultivar, contains the lowest gramine content. The content of gramine in rice resistant cultivars at seedling and tillering stages was ∼2.9-fold and ∼2.4-fold of these in cultivar TN1 rice plants, respectively (Fig. 1).

### Gramine Toxicity to BPHs

**Figure 2:**
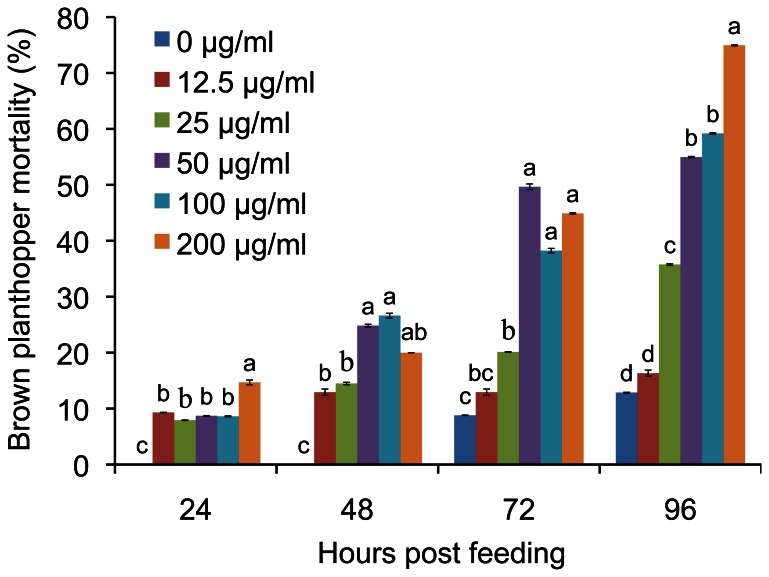
Gramine toxicity to brown planthoppers (BPHs). BPHs were fed on artificial liquid diets containing the indicated concentrations of gramine. At 24, 48, 72 and 96 hrs post feeding (h.p.f.), the BPH mortality was determined. Data represent the means ± SD from three independent experiments with triplicate glass tubes (at least 10 nymphs in each tube) examined for each treatment. Different letters on the bars indicate significance at *P*<0.05.

To determine whether gramine is toxic to rice BPHs, we designed artificial diets containing various concentrations of technical grade gramine (purchased from Kingsley and Keith LTD, UK) and fed BPH nymphs (second to third instar, unless otherwise indicated) with these diets for 24, 48, 72 and 96 hours. Compared to the control diet without gramine, the diets containing gramine significantly increased BPH mortality at 24, 48, 72 and 96 hours post-feeding (h.p.f.). When BPHs were fed diets containing 100 and 200 µg/ml gramine, the mortality of BPHs at 96 h.p.f. was 59.2% and 74.9%, respectively (Fig. 2). BPH mortality and gramine concentration (0–50 µg/ml) displayed a dose-dependent relationship at 72 and 96 h.p.f. (Fig. 2). Therefore, gramine was toxic to BPHs.

### *nlgst1-1* mRNA Level and GST Activity in dsRNA-fed BPHs

If *nlgst1-1* plays a role in detoxification of gramine, then its silencing by RNA interference (RNAi) should reduce the GST activity in BPHs and thus result in increased BPH susceptibility to gramine (see below). To test this possibility, we first generated double-stranded RNA (dsRNA) that targets *nlgst1-1* (Fig. S1, S2, S3). Next, we fed BPHs on artificial diets containing various concentrations of *nlgst1-1* dsRNA and *GFP*-dsRNA (control) for 3 days and confirmed the *in vivo* reduction in transcript level of the targeted transcript by quantitative RT-PCR (qRT-PCR) (Fig. S4A). Finally, we determined the GST activity and gramine sensitivity of BPHs that had been fed on diets containing *nlgst1-1* dsRNA for 3 days.

**Figure 3:**
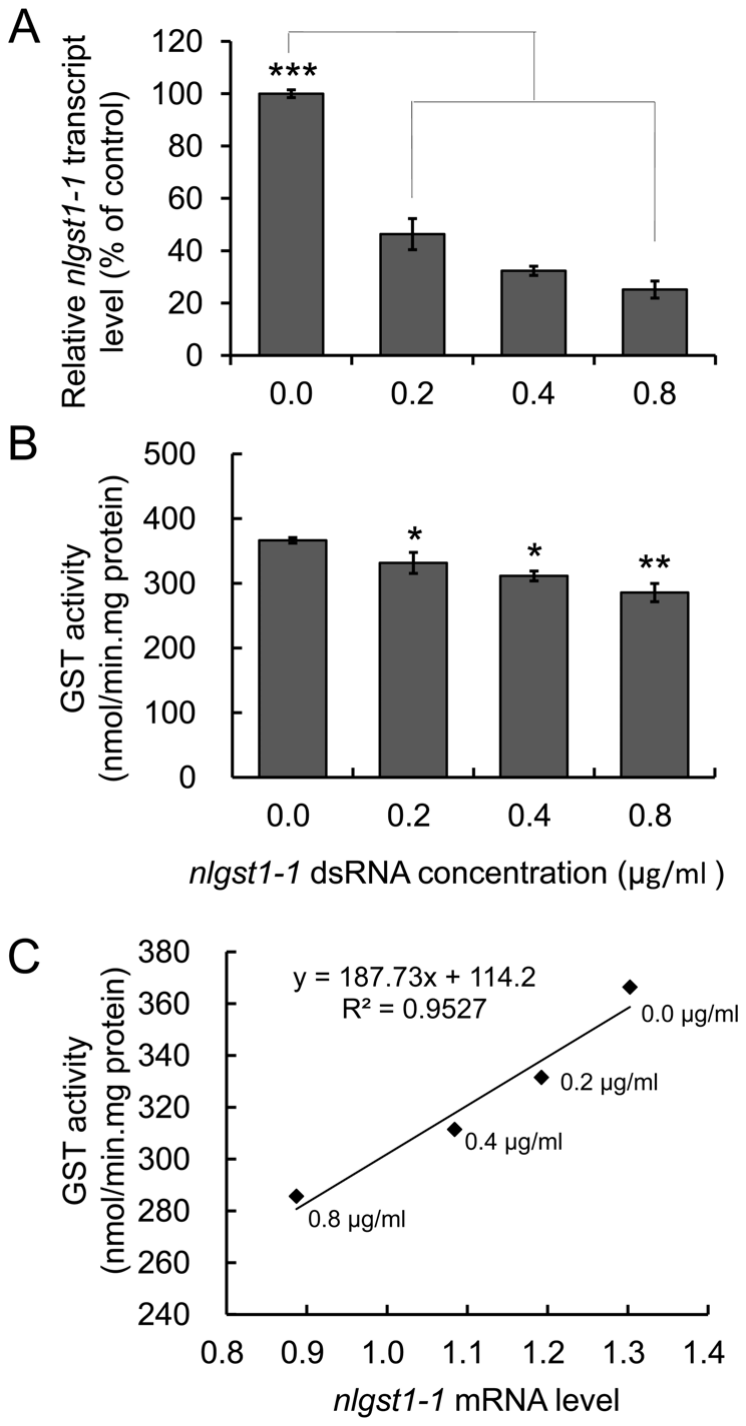
dsRNA-mediated *in vivo* knockdown of GST gene *nlgst1-1* and reduction of GST activities in the dsRNA-fed BPHs. BPHs were first fed on artificial diets containing 0.2, 0.4 and 0.8 µg/ml *^GFP-^*dsRNA or *nlgst1-1* dsRNA for three days. Next, *nlgst1-1* transcript levels in dsRNA pre-treated BPHs were monitored by quantitative real-time PCR (qRT-PCR) at 3 days post feeding. GST activities of dsRNA pre-treated BPHs were also determined by the spectrophotometric approach as described in *Materials and Methods*. All treatments were performed in triplicate, and the experiments were repeated three times. **A**. Reduction of *nlgst1-1* transcript in BPHs fed on diet containing the target dsRNA. **B**. GST activities in BPHs fed on diets with various concentrations of target *nlgst1-1* dsRNA. **C.** Correlation of GST activities with GST gene *nlgst1-1* transcript levels in the dsRNA-fed BPHs. Data represent the means ± SD from three independent experiments. *, **and ***indicates significance at *P*<0.05, *P*<0.01 and *P*<0.001, respectively.

Compared to the relative *nlgst1-1* transcript level of the control BPHs fed the diet containing *GFP*-dsRNA (the relative *nlgst1-1* transcript level of control was normalized as 100%), the relative *nlgst1-1* transcript level in the BPHs fed the diets containing 0.2, 0.4 and 0.8 µg/ml *nlgst1-1* dsRNA was 46.4%, 32.3% and 25.2%, respectively (Fig. 3A). Reduction of BPH *nlgst1-1* transcript level and the concentration of *nlgst1-1* dsRNA in the diets displayed a *dose*-dependent *effect* (Fig. S4B).

To determine whether the observed effects were specific for *nlgst1-1*, we also examined the knockdown effect of *nlgst1-1* dsRNA on the transcript levels of several other unique GST genes in the GenBank, including *GST1* to *GST6*, *GST8* and *GST9* ([26] and Table S1). We first fed BPHs on artificial diets containing various concentrations of *nlgst1-1* dsRNA and *GFP*-dsRNA. Next, we determined the transcript levels of the above-mentioned GST genes at 3 days post feeding on the diets containing *nlgst1-1* dsRNA and *GFP*-dsRNA by qRT-PCR approach. We found that the transcript levels of the other 8 GST genes were not affected when BPHs ingested the diet with *nlgst1-1* dsRNA consistent with their low sequence identities with the *nlgst1-1* sequence (Fig. S5).

As expected, GST activity was significantly lower in BPHs fed on diets containing 0.2, 0.4 and 0.8 µg/ml of *nlgst1-1* dsRNA than BPHs fed a control diet with *GFP*-gene dsRNA. Compared with GST activities in BPHs fed on the diets containing 0.2 and 0.4 µg/ml *nlgst1-1* dsRNA, GST activity in BPHs fed the diet containing 0.8 µg/ml *nlgst1-1* dsRNA was significantly reduced (Fig. 3B), whereas significant differences of the GST activities between the BPHs fed on the diets containing 0.2 µg/ml and 0.4 µg/ml *nlgst1-1* dsRNA was not observed. The *nlgst1-1* dsRNA concentration in the diets and the GST activity in the dsRNA-fed BPHs displayed a linear relationship (Fig. 3C). Furthermore, the *in vivo* reduction of GST activity in the dsRNA-fed BPHs was dependent on the *nlgst1-1* transcript levels (Fig. S6).

### Effect of *nlgst1-1* dsRNA on the Survival of Rice BPHs

**Figure 4:**
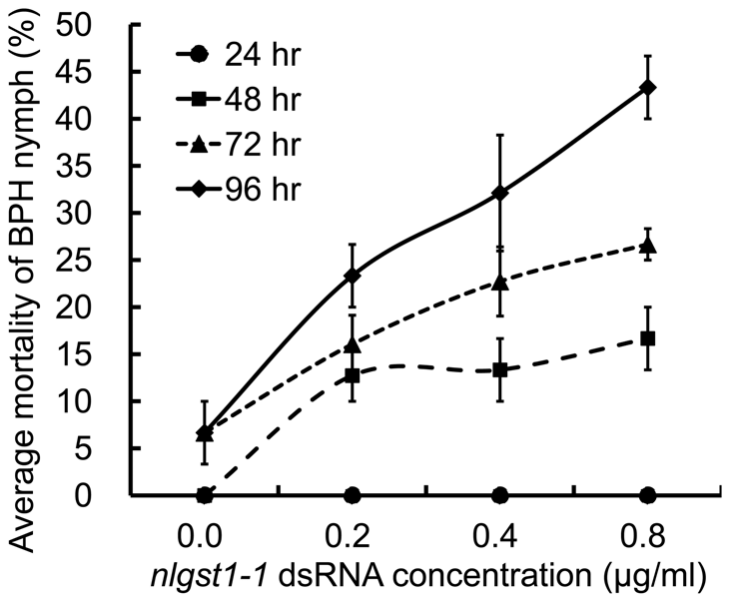
Effect of *nlgst1-1* dsRNA on the mortality of BPH nymphs. *nlgst1-1* dsRNA that targets the knockdown of *nlgst1-1* in BPHs was dissolved in artificial diets at the indicated concentrations (0, 0.2, 0.4 and 0.8 µg/ml), BPH nymphs were then fed on these liquid diets held in the two layers of Parafilm M in controlled growth chambers (90% r.h., 28°C, 14 hr photoperiod) for 96 hrs. Fresh diets containing the indicated concentrations of *nlgst1-1* dsRNA were changed every day. The diet containing 0.8 µg/ml *GFP*-dsRNA was used as control (0 µg/ml *nlgst1-1* dsRNA). The average mortalities of BPHs with the increase of *nlgst1-1* dsRNA concentrations were plotted at 24, 48, 72 and 96 h.p.f. Data represent the means ± SD from three independent experiments with triplicate groups examined for each treatment in each experiment.

To determine the effect of BPH *nlgst1-1* dsRNA mediated *in vivo* depletion of *nlgst1-1* on the survival of BPHs, the insects were fed on diets containing various concentrations of *nlgst1-1* dsRNA for 96 hrs, and the mortality of the dsRNA-fed BPHs was then recorded from 24 to 96 h.p.f. The *nlgst1-1* dsRNA in the diets significantly affected the survival rates of BPHs. The mortalities of dsRNA-fed BPHs at 48, 72 and 96 h.p.f. were significantly higher than control (Fig. 4). At 72 and 96 h.p.f., a significant difference in the mortality of BPHs that had been fed on various concentrations of *nlgst1-1* dsRNA was observed; and the mortality of *nlgst1-1* dsRNA-fed BPHs was significantly correlated with the concentration of *nlgst1-1* dsRNA in the diets (*p*<0.05).

### Sensitivity of the *nlgst1-1* dsRNA-fed BPHs to Gramine

If BPHs detoxify gramine *via* their GSTs, then reduction of the transcript level of BPH GST gene *nlgst1-1* should enhance their sensitivity to gramine. Encouraged by our findings that BPHs feeding on artificial diets containing *nlgst1-1* dsRNA resulted in the *in vivo* reduction of *nlgst1-1* transcript (Fig. 3A), we tested this possibility by determining the responses of the *nlgst1-1* dsRNA pre-fed BPHs to gramine *via* continuously feeding the dsRNA pre-treated BPHs on diets that contained (or lacked) gramine for an additional 3 days. First, we fed BPHs on the diets containing 0 (*GFP*-dsRNA, used as control), 0.2, 0.4 and 0.8 µg/ml *nlgst1-1* dsRNA for 3 days. Next, we divided the dsRNA pre-fed BPHs into two groups, and transferred one group of the dsRNA pre-fed BPHs to the artificial diets containing various concentrations (12.5, 25 and 50 µg/ml) of gramine. We moved another group to the normal control diet without any dsRNA and gramine. The two groups of the dsRNA pre-fed BPHs were simultaneously reared for an additional 3 days for the mortality analysis.

**Figure 5:**
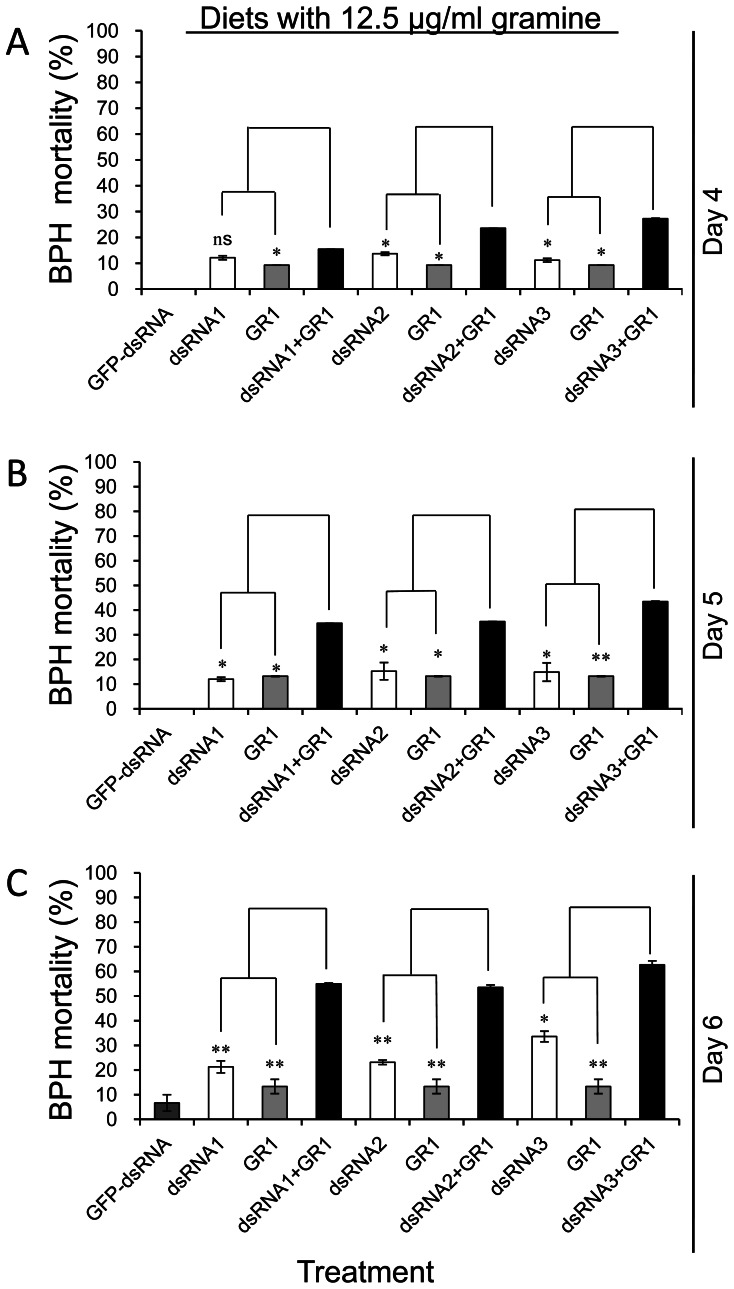
Sensitivity of *nlgst1-1* dsRNA pre-fed BPHs to the diets containing gramine. Gramine was dissolved in artificial diets at the indicated concentrations (GR1 = 12.5 µg/ml). BHPs that had been pre-fed on diets containing 0 (0.8 µg/ml *GFP*-dsRNA, used as control), 0.2 (dsRNA1), 0.4 (dsRNA2) and 0.8 (dsRNA3) µg/ml *nlgst1-1* dsRNA for 3 day were transferred to new diets with the indicated concentrations of gramine and to the normal control diet without dsRNA and gramine. The dsRNA pre-fed BPHs were continually reared on these new diets for an additional 3 days in the controlled growth chambers (90% r.h., 28°C, 14 hr photoperiod). The mortalities of the dsRNA pre-fed BPHs transferred to the new diets were plotted at day 4 (**A**), day 5 (**B**) and day 6 (**C**) post feeding. Data represent the means ± SD from three independent experiments with triplicate groups examined for each treatment in each experiment. *and **indicate significance at *P*<0.05 and *P*<0.01, respectively. “ns” indicates not significant (*P*>0.05).

When BPHs that had been pre-fed the diets containing 0.2, 0.4 and 0.8 µg/ml *nlgst1-1* dsRNA for 3 days were moved to the control diet and to the diets containing 12.5 µg/ml gramine, at day 4 (24 hrs after being placed on new diets) post feeding, the mortality of the *nlgst1-1* dsRNA pre-treated BPHs that were fed the diets containing gramine was significantly higher than control (*GFP*-dsRNA pre-fed BPHs reared on the diets with the same concentrations of gramine) and than the *nlgst1-1* dsRNA pre-treated BPHs that were placed on normal diet without dsRNA and gramine (Fig. 5A). At day 5 and day 6 post feeding, the mortality of the *nlgst1-1* dsRNA pre-fed BPHs that were placed on the diets containing gramine dramatically increased when compared to control and to the *nlgst1-1* dsRNA pre-fed BPHs that were placed on a normal diet (Fig. 5 B, C). When BPH nymphs that had been pre-fed with 0.8****µg/ml *nlgst1-1* dsRNA for 3 days and then placed on diets containing 12.5 µg/ml gramine, at day 6 post feeding, the mortalities of the nymphs were up to 62.7±1.6% (Fig. 5C). Similar results were observed when the *nlgst1-1* dsRNA pre-fed BPHs were fed on diets containing 25 and 50 µg/ml gramine (Fig. S7).

When BPHs that had been pre-fed *nlgst1-1* dsRNA for 3 days were placed on diets with gramine for an additional 3 days, the mortalities of the *nlgst1-1* dsRNA pre-fed BPHs at day 4, day 5 and day 6 post feeding were significantly correlated with the concentrations of gramine in the diets (Fig. S8).

BPHs that had been placed on diets containing *nlgst1-1* dsRNA for 3 days were transferred to diets containing various concentrations of gramine for an additional 3 days, the mortality of the dsRNA pre-fed BPHs rapidly increased with the increase of gramine concentration (Fig. S8). The survival of *nlgst1-1* dsRNA pre-fed BPHs was thus affected by both *nlgst1-1* dsRNA and gramine concentration in the diets. The mortality of *nlgst1-1* dsRNA pre-fed BPHs at day 4 to day 6 post feeding was significantly affected by *nlgst1-1* dsRNA or gramine in the diets (p<0.01), and a significant interactive effect of *nlgst1-1* dsRNA and gramine on BPH mortality was found at day 4 post feeding (p<0.01) (Table 1).

**Table 1:** Effect of brown planthopper **(**BPH, *Nilaparvata lugens* **)** *nlgst1-1* dsRNA and gramine in artificial diets on BPH mortality.

Source	df	4 d.p.f.		5 d.p.f.		6 d.p.f.	
		F	P value	F	P value	F	P value
dsRNA	2	42.850	<0.01	19.955	<0.01	22.260	<0.01
Gramine	3	80.379	<0.01	76.694	<0.01	163.138	<0.01
dsRNA×Gramine	6	9.938	<0.01	1.752	0.152	0.707	0.647
Total	36						

Note: d.p.f., days post feeding.

### *nlgst1-1* dsRNA-fed BPHs were Less Adaptive to their Host Plants

**Figure 6:**
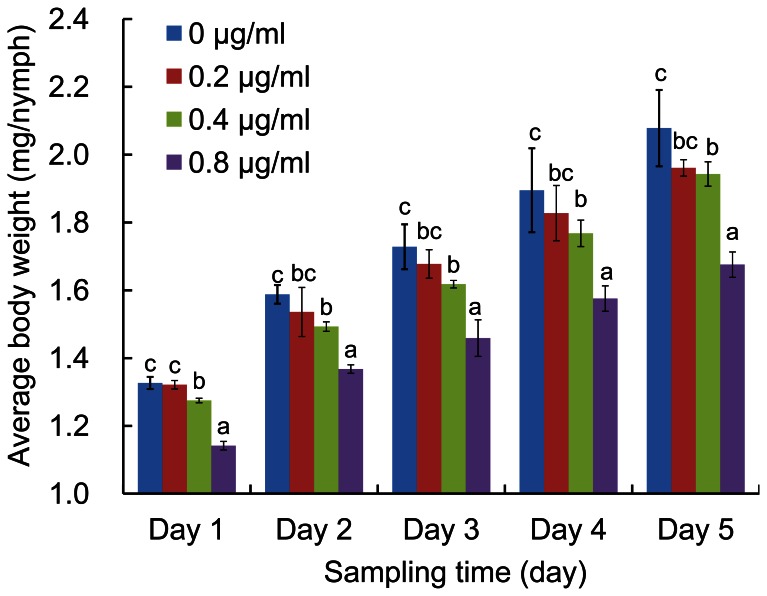
Effect of *nlgst1-1* dsRNA on the body weight grain of BPH nymphs. BPH nymphs that had been pre-fed on artificial diets containing the indicated concentrations of *nlgst1-1* dsRNA for 3 days were transferred to cultivar TN1 (BPH susceptible rice variety with low gramine content) rice plants to continually be reared for an additional 5 days in the controlled growth chambers (90% r.h., 28°C, 14 hr photoperiod). The body weight of each BPH nymph was weighed every day during this 5-day period of rearing on the rice plants. The average weight of *nlgst1-1* dsRNA pre-fed BPHs was plotted as a function of days post rearing on the rice plants. All treatments were performed in triplicate groups with at least 10 nymphs in each group. The experiments were repeated at least five times. Data represent the means ± SD from at least five independent experiments. Bars with different letters indicate significance at *P*<0.05.

Encouraged by our findings that (1) rice plants contain gramine (Fig. 1), (2) oral delivery of *nlgst1-1* dsRNA effectively reduced *nlgst1-1* transcript levels and GST activity in BPHs *in vivo* (Fig. 3 A, B), and (3) BPHs that had been placed on diets containing *nlgst1-1* dsRNA became more sensitive to gramine (Fig. 5, S7), we hypothesized that if BPHs detoxify gramine from rice plants *via* their GSTs, then interference of BPH *nlgst1-1* should reduce their adaptability to rice plants containing gramine. To interrogate this hypothesis, we first fed BPHs artificial diets containing various concentrations of *nlgst1-1* dsRNA that targeted the knockdown of BPH *nlgst1-1* for 3 days. Next, we transferred the dsRNA pre-fed BPHs to cultivar TN1 rice plants, which contain low levels of gramine (0.26 mg/g.dw gramine), and then continuously reared the insects on the rice plants for an additional 5 days while weighing each BPH nymph every day. Finally, we evaluated the adaptability of the *nlgst1-1* dsRNA-fed and control BPHs to cultivar TN1 rice plants based on the average body weight gain during an additional 5-day period of rearing on the rice plants. When BPH nymphs that had been pre-fed on the diets containing higher concentrations of *nlgst1-1* dsRNA (> = 0.4 µg/ml) were transferred to cultivar TN1 rice plants, their daily body weight and body weight gain after rearing on the rice plants was significantly inhibited when compared to controls (Fig. 6, S9).

## Discussion

During the co-evolution of plant and herbivorous insects, the mechanisms of plant defense and insect counter-defense are synergistically developed [27], [30]. Plant secondary metabolites such as phenolics, terpenoids and nitrogen-containing metabolites are generally involved in plant defense against insect attacks [17], [31], [32], and adversely affect the feeding behavior and survival of insects [27], [33]. Gramine is a simple alkaloid that exists in some terrestrial gramineous crops and confers defense against aphids [14], [29], [34]. Our data demonstrate that gramine also exists in hydrophytic plants such as rice, and its content is increased in rice plants with higher levels of BPH resistance (Fig. 1). These findings suggest that accumulation of high concentrations of gramine in rice cultivars may contribute to defense against insect pests such as BPHs. To the best of our knowledge, this is the first report about rice plants containing gramine and its relationship with BPH resistance.

Gramine has been considered to be an antagonistic agent that blocks the growth, development and survival of some sap-sucking insects such as greenbug (*Schizaphis graminum*), corn aphid (*Rhopalosiphum maidis*) and grain aphid (*Sitobion avenae*) [17], [35], [36]. In this study gramine was found to be toxic to BPHs, and gramine content within rice plants was highly related to the resistance of rice varieties to BPHs (Fig. 1). These findings therefore further demonstrate that gramine plays an important role in rice defense against BPHs. However, plant defense against herbivorous insects involves a wide variety of plant secondary metabolites [16], [29], [37], [38]. Therefore, the involvement of other secondary metabolites including phenolics, non-protein amino acids and other alkaloids, in rice resistance to BPHs should be further investigated to elucidate the mechanisms of rice defense against BPHs.

BPHs mainly feed on rice phloem fluid. However, a reasonable and/or practicable approach to detect the exact concentration of gramine in rice phloem fluid is still unavailable. Therefore, in this work, gramine content in rice phloem fluid was estimated from the concentration in total rice leaves as the previously described methods [15], [37], [39], [40].

To *in vitro* investigate the toxicity of a compound derived from plant metabolites on sap-sucking insect such as aphids and BPHs, a wide range of concentrations (0.003∼0.1% W/W) of the compound are usually incorporated into artificial diets on which the tested insets are fed [17], [41], [42]. It is noted that gramine concentrations in a portion of the artificial diets in our experiments may be much higher than the estimated gramine concentrations in rice phloem fluid. However, to make sure that gramine works inside the insects and quickly yields measurable toxic effect on the tested BPHs, high concentrations of the compound in the artificial diets are necessary [17], [41], [42], since the toxicity of gramine may be undetectable or not easy to detect if gramine concentration in the diets is as low as that in plant tissues. Therefore, to obtain the measurable toxic effect of gramine on BPHs, we incorporated a wide range of gramine concentrations (0 to 200 µg/ml) in the artificial diets on which BPHs were fed. Our findings demonstrate that gramine is toxic to BPHs because no matter how low or high gramine concentration is in the diet, diets with gramine have significant effect on BPHs’ survival (Fig. 2). Further study is needed to clarify other effects of gramine on BPHs, including antifeedant, growth suppression, population development, etc., with lower gramine concentrations (roughly equal to the estimated gramine concentration in the phloem fluid of BPH resistant rice plants).

Herbivorous insects have developed adaptability to their host plants *via* overcoming the toxic metabolites in plants during herbivore and plant co-evolution. To adapt themselves better to hosts containing deleterious secondary metabolites, some insect pests employ their detoxification enzymes, including cytochrome P450 monooxygenases and GSTs, to disarm plant cytotoxic metabolites [39], [43], [44]. GSTs are not only important detoxification enzymes that catalyze the conjugation of electrophilic substrates to glutathione, but also display peroxidase and isomerase activities [45]. For the BPH defense system, the tissue-specific transcript levels of detoxification-related genes may reflect the functional diversification of these enzymes. In BPHs, GST gene *nlgst1-1* displayed higher transcript levels in the insect intestine in comparison to P450 genes [28], suggesting that toxic metabolites from plants could be first oxidized and detoxified by GSTs in the intestine of BPHs. Our results further support the detoxification effect of GST in BPHs on toxic metabolites from host plants and insects adapt themselves to various host plants *via* their detoxification machinery.

A total of nine GST genes were identified in BPH [26]. Of these, only *nlgst1-1* was previously shown to play an important role in BPH resistance to insecticides [18], [20]. The functions of the other 8 GST genes in BPHs remain obscure, although their orthologues are known to play a role in insecticide detoxification in other insect species [46]. From the view of coevolution, insects including BPHs employ their GSTs to detoxify plant toxic compounds and adapt to the plants that contain the toxic compounds but not to metabolize insecticides, since the GSTs have existed long before the insecticides have. Thus, the primordial functions of insect GSTs are to metabolize toxic compounds from their host plants [47]. Our data indicates that dsRNA that targets the knockdown of BPH *nlgst1-1* significantly down-regulated *nlgst1-1* expression level in BPHs, but had little effect on the transcript levels of the other eight GST genes due to their low sequence identities with that of *nlgst1-1* (Fig. S5), suggesting that targeting of BPH *nlgst1-1* dsRNA to its corresponding gene is highly specific and effective. In addition, the knockdown efficiency of BPH *nlgst1-1* by our approach of oral delivery of the target dsRNA reached 75%, however, the GST activity did not dramatically reduce when BPH *nlgst1-1* was depleted *in vivo*. Our findings suggest that the other GST genes can complement the targeted GST’s function somewhat. Therefore, further work is needed to clarify the contribution of the other eight GST genes in BPHs in the detoxification of secondary metabolites such as gramine in rice plants.

Besides GSTs, other enzymes in herbivorous insects such as esterases, acetylcholinesterases and cytochrome P450 monooxygenases also defend against plant-derived deleterious/toxic secondary metabolites [37], [48]. Although significant changes in the activities of esterase and mixed functional oxidases (MFOs) in BPHs were not detected when the BPHs were fed on resistant and susceptible rice varieties for one generation [49], further clarification of the involvement of these enzymes in BPHs in metabolism of gramine is warranted.

Microinjection and ingestion have proven effective approaches to deliver dsRNAs, small interference RNAs (siRNAs), or short hairpin RNAs (shRNAs) of target genes into insects, thereby enabling *in vivo* studies of gene functions in various systems, including *BtSnap* in silverleaf whitefly (*Bemisia tabaci*) [50], *c002* in pea aphid (*Acyrthosiphon pisum*) [51] and *Calreticulin* in BPH (*N. lugens*) [52]. In the present study, we employed the approach of the combination of *nlgst1-1* dsRNA and gramine from plants, by which efficiently depleted expression of the target gene *nlgst1-1* in BPHs largely increased BPH sensitivity to gramine and reduce BPH adaptability to gramine-containing plants. Compared with other approaches to deliver dsRNAs to the target insects, the amounts of target dsRNA required in our oral delivery system is significantly lower than previously reported ([50], [52], [53] and this study). Our approach may therefore enable further studies into molecular approaches to controlling sap-sucking insects such as BPHs.

It is noted that the significant interactive effect of *nlgst1-1* dsRNA and gramine on the mortalities of BPHs that had been pre-fed on diets containing *nlgst1-1* dsRNA for 3 days and then placed on diets with gramine was not observed at day 5 and day 6 post feeding (Table 1). This unexpected result could be affected by (1) the limited duration of gene knockdown in insects which could result in the gradual recovery of the interfered target genes when the insects are no longer fed on the target dsRNAs [54]–[56]; (2) the main contribution of gramine toxicity to BPH mortalities which resulted in less difference between the treatments of BPHs that had been fed on diet containing *GFP*-dsRNA and then placed on diet with gramine (GR3) and BPHs that had been fed on diets containing *nlgst1-1* dsRNA and then placed on diet with gramine (dsRNA X+GR3, where X = 1, 2, 3) (Fig. S7C, right panel). This unexpected result suggests that in the practice of controlling BPH population, BPHs are continually fed on diets with *nlgst1-1* dsRNA should be more beneficial to the interactive effect of *nlgst1-1* dsRNA and gramine, even in lower concentration of gramine.

It is also noted that the treatment of *nlgst1-1* dsRNA results in relatively high lethal effects on BPHs. *nlgst1-1* is the only GST gene known to plays an important role in BPH detoxification of endogenous and exogenous toxic compounds [18], [47]. Depletion of *nlgst1-1* may reduce GST clearance of toxic compounds from BPH’s metabolites, which leads to mortality. In addition, artificial diets are usually less toxic or even non-toxic to insects mainly because insects have a detoxification system that completely metabolizes dietary compounds. However, if a key factor of the detoxification system such as GST is depleted, the ingested compounds cannot be effectively metabolized in the intestinal tract or the intermediates cannot be effectively decomposed, which may result in the accumulation of some toxic metabolites. These metabolites may cause BPH mortality when their concentrations accumulate to toxic levels.

*In planta*, the expression of dsRNAs that cause the knockdown of functional genes in chewing insects provides a new avenue for environmentally-friendly pest management [43], [57]. Furthermore, the knockdown of functional genes by dsRNA-transgenic plant-mediated RNA interference is also achieved in hemipteran insects such as aphids and brown planthoppers [58], [59]. Our findings indicate that GST gene *nlgst1-1* is essential for BPH gramine detoxification and adaptation to gramine-containing host plants, thus BPH *nlgst1-1* gene is a potential target for controlling BPH population in the practice of agriculture. However, during the co-evolutions of herbivorous insects and host plants, the risk of insect resistance to RNAi strategies by the acquisition of mutations in the target genes such as GST genes should also be considered.

## Materials and Methods

### Plant and Insect

Rice cultivars, ASD7 and IR36 (resistant to BPHs), IR56 and Rathu Heenati (highly resistant to BPHs), and Taichung Natuva 1 (TN1, susceptible to BPHs) [12], [60], [61] were grown in net house at the Science Park of South China Agricultural University, Guangzhou, Guangdong province, China. For every cultivar, 20 seeds were sown in polypots (30×20×6 cm) in triplicate. White nylon mesh was used to protect the rice plants from insect attacks and mechanical damage during rice growth. Rice plants at seedling and tillering stages were collected for gramine content assay.

Rice BPHs (biotype II) were reared on susceptible cultivar TN1 rice seedlings in controlled environmental growth chambers [90% relative humidity (r.h.), 28°C, 14 hr light/10 hr dark, unless otherwise indicated]. All experimental insects were allowed to reproduce at least one generation for adapting new condition before they were used in the subsequent experiments.

### Gramine Content Assay

A total of 10 rice tillers from each polypots were randomly sampled at seedling and tillering stages for gramine content assay. Preparation of samples and gramine extraction were carried out as previously described using high-performance liquid chromatography (HPLC) method [29], [62] with a minor modification. Briefly, 1.0 gram dry powder of sample was homogenized in 30 ml of 0.01% acetic acid (100∶0.01, v/v). The extract was filtered through glass wool and adjusted to pH 9.15 with concentrated NH_4_OH. The resultant mixture was centrifuged (Himac CR22E, Hitachi Koki Co. Ltd. Japan) at 1000× *g* for 5 min at 25°C. Ten ml of supernatant was added into a Sep-Pak column (Waters, MA, USA), and sequentially eluted by flowing 2 ml of methyl cyanide, 2 ml of KH_2_PO_4_ (0.001 M, pH 7), 2 ml of a KH_2_PO_4_ (0.05 M, pH 9.5) and isopropyl alcohol mixture (70∶30, v/v), 2 ml of a KH_2_PO_4_ (0.05 M, pH 9.5) and isopropyl alcohol mixture (95∶5, v/v) and 1.5 ml of a KH_2_PO_4_ (0.05 M, pH 2.3) and isopropyl alcohol mixture (70∶30, v/v). The resultant eluate of each sample was evaporated to 1 ml by N_2_ and used for HPLC analysis of gramine content.

Gramine content assay was performed on a Milton Roy**/**LDC HPLC (Milton Roy, FL) equipped with a C_18_ reversed-phase column and a 250-mm×4.0-mm, 5-µm particle size C_18_ column (Thermo Fisher Scientific Inc. USA). Ten µl of the concentrated eluate was injected with an auto-injector (Spark Holland Inc., Holland). Eluent was the mixture of 0.025 M KH_2_PO_4_ and 0.1% triethylamine (pH 7.15) and methyl cyanide (60∶40, v/v). The eluate was analyzed at 221 nm with a Milton Roy UV SM 4000 detector (Milton Roy, FL, USA) at a flow rate of 0.5 ml/min at 25°C column temperature. The retention time for gramine was 7.5 min. The response factor and linearity were determined with technical grade gramine purchased from Kingsley and Keith LTD (UK).

### Bioassay of Gramine Toxicity to BPHs

To determine the gramine toxicity to BPHs, technical grade gramine was incorporated into artificial diets. Artificial diets for BPHs were prepared according to previously described methods [63], [64] with a minor modification. Briefly, technical grade gramine was dissolved in liquid artificial diets at the concentrations of 0, 12.5, 25, 50, 100 and 200 µg/ml. Two layers of the stretched Parafilm M (Pechiney Plastic Packaging Company, Chicago, USA) with liquid diet were placed in a transparent cylindrical glass tube (Ф = 2.5 cm, length = 10 cm) and covered with gauze on the top to allow air and humidity exchange. At least ten BPH nymphs in a group (triplicate groups were tested for a treatment in each experiment, unless otherwise indicated) were fed on the liquid diets held between two layers of Parafilm M. The experiment was repeated three times. BPHs were cultured in the controlled growth chambers. Diets and feeding sachets were changed daily to avoid diet deterioration and microbial contamination. The BPH mortality was determined at 24, 48, 72 and 96 h.p.f.

### Generation of dsRNA Targeting to the Knockdown of BPH *nlgst1-1*

Total RNA of BPHs stored in liquid nitrogen was extracted using RNeasy Mini Kit (QIAGEN, Hilden, Germany) from the 3rd BPH instars. The potential genomic DNA contamination was eliminated by a treatment with DNase I (NEB, Ipswich, USA) after the RNA extraction procedure. RNA concentration and quality were determined using a Nanodrop spectrophotometer (Thermo Scientific, USA). cDNA mixture was reverse synthesized from 2 µg of total RNA using reverse transcriptase (QIAGEN, Hilden, Germany) and oligo (dT)_15_ (TIANGEN, Beijing, China ) as primer. Full-length cDNA of BPH *nlgst1-1* was isolated by PCR approach with specific primers (5′-ATGCCAATTGATCTGTACTACGTACC-3′ and 5′-TTACTTTCCGGTCATAGCCTTGAAC-3′, forward and reverse primer, respectively). Amplified PCR products were ligated into the pDrive cloning vector (QIAGEN, Hilden, Germany). The resultant pDrive-*nlgst1-1* plasmid was transformed into Trans1-T1 Phage Resistant Chemically Competent Cell (TransGen Biotech, Beijing, China). Positive clones were identified by the approaches of PCR detection and plasmid digestion with restriction endonucleases *Mlu*I and *Nhe*I (NEB, Ipswich, USA). BPH *nlgst1-1* cDNA insertion was further confirmed by DNA sequencing.

pDrive-*nlgst1-1* amplified cDNA fragments flanked by T7 polymerase promoter sequences were used as the template for *in vitro* synthesis of *nlgst1-1* dsRNA. Forward and reverse primers used to amplify the template were 5′-TAATACGACTCACTATAGGGAAAGCT-3′ and 5′-TAATACGACTCACTATAGGGCCTAGGCTCGAGAAGCT-3′, respectively. PCR products were examined on 1% agarose gel for verification and then purified using TIANgen Midi Purification Kit (TIANGEN, Beijing, China). A dsRNA that targets BPH *nlgst1-1* expression was then *in vitro* generated using HIScribe™ T7 *In Vitro* Transcription Kit (NEB, Ipswich, America) and purified with RNA Clean Kit (TIANGEN, Beijing, China). Purified BPH *nlgst1-1* dsRNA was stored at −80°C until use. A dsRNA that targets the green fluorescent protein (GFP) gene [*Aequorea coerulescens* (belt jellyfish), AY151052.1] expression was also produced as described above and used as control.

### BPH Response to Diets Containing *nlgst1-1* dsRNA

To analyze the knockdown effect of *nlgst1-1* dsRNA on BPH *nlgst1-1* transcript level, GST activity as well as the mortality of *nlgst1-1* dsRNA-fed BPHs, *nlgst1-1* dsRNA was dissolved in artificial diets at the concentrations of 0.2, 0.4 and 0.8 ^µg/ml^, and BPH nymphs were fed on these *nlgst1-1* dsRNA-containing liquid diets held between two layers of Parafilm M. Diets containing various concentrations (0.2, 0.4 and 0.8 ^µg/ml^) of *GFP*-dsRNA were used as control. The BPH nymphs were cultured in controlled growth chambers. Diets and feeding sachets were changed daily to avoid diet deterioration and microbial contamination. Two experiments were simultaneously conducted to test BPH response to diets containing *nlgst1-1* dsRNA. The first experiment was designed to determine the daily mortality rate of dsRNA treated BPHs. Briefly, BPH nymphs were fed on diets containing various concentrations of *nlgst1-1* dsRNA and *GFP*-dsRNA, and the numbers of dead nymphs from each treatment were recorded at 24, 48, 72 and 96 h.p.f. to calculate the daily mortality rate of dsRNA-fed BPHs. The second experiment was designed to evaluate the knockdown effect of *nlgst1-1* dsRNA. In this experiment, BPH nymphs that had been fed on diets containing various concentrations of *nlgst1-1* dsRNA and *GFP*-dsRNA for 3 days were collected and stored in liquid nitrogen for analyzing *nlgst1-1* transcript levels with quantitative real-time PCR (qRT-PCR) approach (see below) and GST activities. The experiments were repeated at least three times.

### qRT-PCR and Semi-quantitative RT-PCR (sqRT-PCR) Analyses

Total RNA of dsRNA pre-fed BPH nymphs was extracted as described above. qRT-PCR was performed as the previously described standard curve method [65]. A total of 20 µl reaction volume contained 10 ng of cDNA, 0.1 µM each gene specific primers and 10 µl SYBR® Premix Ex Taq™ (TaKaRa, Japan). Reactions were performed in a Thermal Cycler Dice Real Time System ABI 7500 (Applied Biosystems). Reaction conditions were as follow: an initial incubation of 50°C for 2 min and 95°C for 30 sec; 40 cycles of 95°C for 5 sec and 60°C for 34 sec. A dissociation step cycled at 95°C for 15 sec, 60°C for 1 min, 95°C for 30 sec and 60°C for 15 sec. Standard curves were obtained using serial grade dilution of pooled total cDNAs from 50 individuals. The primers of BPH *nlgst1-1* used in the qRT-PCR assay were: 5′-CTAAGGTCAACCAACGCCTCTACT-3′ (forward primer) and 5′-GATGTTCTTGTATGGGCTCAGGTC-3′ (reverse primer), which produced a 266 bp band. The forward and reverse primers for the internal control (β-actin) were 5′–TGGACTTCGAGCAGGAAATGG-3′ and 5′-ACGTCGCACTTCATGATCGAG-3′, respectively [66], which produced a 200 bp band. The standard curve method [65] was used to evaluate the quantitative variation. To clearly demonstrate the knockdown effect of *nlgst1-1* dsRNA, *nlgst1-1* transcript level in *GFP-*dsRNA fed BPHs (control) was regarded as 100%. The knockdown efficiency in *nlgst1-1* dsRNA-fed BPHs was calculated as the following: *nlgst1-1* dsRNA mediated *nlgst1-1* knockdown efficiency  = 100% − relative percentage of *nlgst1-1* transcript level in *nlgst1-1* dsRNA fed BPHs.

To evaluate whether *nlgst1-1* dsRNA impacts the transcript levels of the other 8 GST genes in rice BPHs that were fed on diets containing various concentrations of *nlgst1-1* dsRNA, the transcript levels of the 8 GST genes were also determined by qRT-PCR following the reaction conditions described above. The primers used in the qRT-PCR analysis are listed in Table S1.

To further validate the reduction of *nlgst1-1* transcript levels in *nlgst1-1* dsRNA-fed BPHs, sqRT-PCR was also conducted. After a 3-day period of feeding on diets containing *nlgst1-1* dsRNA, the survival nymph samples were quickly frozen in liquid nitrogen. Extraction of total RNA from the frozen nymph samples and the synthesis of the first strand cDNA were performed as described above. sqRT-PCR was performed using 50 ng of cDNA as template and 400 nM final concentration of each primer under the following conditions: a 5-min initial denaturation at 94°C; 20 amplification cycles (denaturation at 94°C for 30 s, annealing at 55°C for 30 s, and extension at 72°C for 30 s); and a final extension at 72°C for 7 min. The transcript level of endogenous *β*-actin gene was used as internal control. The PCR reaction was performed by using 2×Taq PCR Master Mix (Bio-Republic, Beijing, China). The primers used to amplify the fragments of *nlgst1-1* and *β*-actin gene in the sqRT-PCR were the same as those used in the qRT-PCR assay.

### GST Activity Assay of dsRNA-fed BPHs

GST activity assay was performed as the previously described method [67] with a minor modification. Briefly, about 50 mg of the *nlgst1-1* dsRNA and *GFP*-dsRNA fed BPHs frozen in liquid nitrogen was homogenized in 1 ml of 0.1 M PBS buffer (pH 6.5) at 4°C. The homogenate was centrifuged at 13,000 *g* for 15 min at 4°C, and the supernatants were used to analyze GST activity. The reaction mixture contained 0.92 ml of the above-mentioned PBS, 20 µl of 50 mM 1-Chloro-2, 4-dinitrobenzene (CDNB) and 20 µl of 50 mM reduced glutathione. Finally, thirty microliter of the supernatant was quickly added into the reaction mixture, the change of the absorption at 340 nm was monitored with SP-756P spectrophotometer (Shanghai Metash Instruments Co. Ltd., China).

Total protein concentrations in the above-mentioned supernatants were determined using the previously described method [68]. Absorbance of the reaction mixture was read at 595 nm with a spectrophotometer and protein concentration was determined from a standard curve established using the known concentration of bovine serum albumin (Sigma, Louis, USA) and the protein assay reagent.

### Sensitivity of *nlgst1-1* dsRNA fed BPHs to Gramine

To evaluate the sensitivity of dsRNA-fed BPHs to gramine, BPH nymphs were first fed on diets containing 0, 0.2, 0.4 and 0.8 ^µg/ml^ of *nlgst1-1* dsRNA for three days as described above. BPHs fed on diets containing 0.8 ^µg/ml^ *GFP*-dsRNA were used as control (0 ^µg/ml^ of *nlgst1-1* dsRNA). Next, all the survival dsRNA-fed BPH nymphs were used to test their sensitivity to gramine. The liquid artificial diets contained 0, 12.5, 25 and 50 µg/ml of gramine. The *nlgst1-1* dsRNA and *GFP*-dsRNA fed BPH nymphs were fed on new diets with various gramine concentrations but without *nlgst1-1* dsRNA. In each treatment, at least 10 dsRNA-fed BPH nymphs in a group (for each treatment, triplicate groups were tested in an experiment) were transferred and fed on the new diets with or without gramine for an additional 3 days under the same controlled conditions as described above. The experiment was repeated three times. The dead individuals of the *nlgst1-1* dsRNA and *GFP-*dsRNA fed BPH nymphs were recorded at 4, 5 and 6 days (24, 48 and 72 hrs after being placed on the new diets, respectively) post feeding for the mortality assay.

### Adaptation Assay of *nlgst1-1* dsRNA fed BPH Nymphs to Host Plants

Cultivar TN1 rice seedlings were cultured using hydroponic culture solution to 4–5-leaf stage [69], the seedlings were then transplanted to test tubes (Ф = 2.5 cm, length = 25 cm) with hydroponic culture solution that allows the seedlings to continually grow. The seedlings in the test tubes were supported by absorbent cotton around the seedling bases, 1–2 seedling plants were planted in each test tube. The BPH nymphs that had been pre-fed on diets containing *nlgst1-1* dsRNA and *GFP-*dsRNA (control) for 3 days were transferred and reared on rice seedlings growing in the test tubes for an additional 5 days. The top of the test tube was covered with gauze to allow air and humidity exchange. New rice seedlings and hydroponic culture solution in the test tubes were exchanged every 2 days to provide enough nutrition for the nymphs and rice seedling growth. During a 5-day period of being reared on cultivar TN1 rice plants, the dsRNA pre-treated BPH nymphs were weighted on an electronic balance (Shanghai Taiheng Instruments Co. Ltd., China) every day. The body weight gain was then calculated every day for evaluating the responses of *nlgst1-1* dsRNA treated BPHs to host rice plants. The experiment was conducted in the controlled environmental growth chambers, and the experiment was repeated at least five times.

### Statistical Analysis

Toxicity of gramine to BPHs was subjected to significant analysis using the PriProbit Program V1.6.3 [70]. Analysis of variance (ANOVA) was performed with the SPSS program (SPSS 13.0). All the correlational relationships were also analyzed by the SPSS program. The Least Significant Difference (LSD) is used to indicate the significance of the treatments. To increase homogeneity of the variances, all data of BPH mortalities were transformed into the values of square root before analysis. A P-value of <0.05 was considered as significant difference.

## Supporting Information

Figure S1 **Sequence information of pDrive-** ***nlgst1-1*** **.** T7 RNA polymerase promoter (within frame): bases 1–20 and 764–783; multiple cloning sites (underline): bases 28∼78 and 730∼763; *nlgst1-1* ORF region (in gray): bases 79–729. pDrive-*nlgst1-1* flanked by T7 polymerase promoter sequences was used as the template for *in vitro* synthesis of *nlgst1-1* dsRNA. Forward and reverse primers used to amplify *nlgst1-1* cDNA template were 5′-TAATACGACTCACTATAGGGAAAGCT-3′ (bases 1-26 ) and 5′-TAATACGACTCACTATAGGGCCTAGGCTCGAGAAGCT-3′ (bases 747–783 ), respectively.
(TIF)

Figure S2 **Deduced amino acid sequence of nlgst1-1 protein.** Asterisks (below) indicate GSH binding site (G-site) (11, 52.54, 66.67); black dots (below) indicate substrate binding pocket (H-site) (103, 107…108, 111…112, 115,119, 164, 167, 206). The underlined amino acid residues indicate C-terminal domain interface (9.10, 12, 15, 19, 22.23, 68, 71). The double-underlined amino acid residues indicate N-terminal domain interface (99, 102.103, 156, 159, 163, 167, 199, 202, 206). A horizontal dash indicates terminator codon.
(TIF)

Figure S3 ***In vitro*** **generation of dsRNA that targets the knockdown of brown planthopper (BPH) glutathione S-transferase (GST) gene** ***nlgst1-1*** **.** Agarose gel (1%) electrophoresis of *nlgst1-1* cDNA (PCR amplification product) (**A**), *in vitro* transcribed DNA template using pDrive- *nlgst1-1* plasmid as templates (**B**), and *in vitro* transcribed *nlgst1-1* dsRNA (**C**). The expected bands are indicated in lane 1 in panels (**A**) and (**B**), and in lane 2 and lane 3 in panel (**C**) with arrows. Lane 2 in panels (**A**) and (**B**) and Lan1 in panel (**C**) are DNA molecular weight (MW) markers; the numbers indicate the sizes (bp) of the bands in the markers.
(TIF)

Figure S4 **dsRNA-mediated knockdown of** ***nlgst1-1*** **transcript in BPHs fed with diets containing the indicated concentrations of** ***nlgst1-1*** **dsRNA.** A: Semi-quantitative RT-PCR products (*nlgst1-1* transcript) from rice BPH nymphs fed on diets containing various concentrations of the targeted dsRNAs. Lane 1 to lane 4 represents *nlgst1-1* transcript from BPH nymphs fed on the diets containing 0 (0.8 µg/ml *GFP-*dsRNA, used as control), 0.2, 0.4 and 0.8 µg/ml *nlgst1-1* dsRNA, respectively. B: correlation of *nlgst1-1* transcript level with the concentration of *nlgst1-1* dsRNA (P<0.05).
(TIF)

Figure S5 ***nlgst1-1*** **dsRNA has no effect on other GST-related genes in rice BPHs.** The BPHs were fed with the diets containing 0.2 µg/ml (**A**), 0.4 µg/ml (**B**) and 0.8 µg/ml (**C**) *nlgst1-1* and *GFP*-dsRNAs. The dsRNA-fed PBHs were then used to analyze *nlgst1-1* transcript level by qRT-PCR approach as described in the text. The detailed information about *GST1* to *GST9* was shown in Table S1 and sequence homology (%) among the GST genes was compared with DNAMAN software and shown in panel **D**. The homology of *nlgst1-1* and *GST1-GST6*, *GST8* and *GST9* is 9.51%, 21.72%, 32.90%, 16.74%, 34.36%, 20.11%, 33.87% and 34.67%, respectively. The *nlgst1-1* transcript level in *GFP*-dsRNA fed BPHs was used as control [*GFP* (Ctrl)] and normalized as 1. Data represent the means ± standard deviation from three independent experiments. * and ** indicates significance at *P*<0.05 and *P*<0.01, respectively.
(TIF)

Figure S6 **Correlation of** ***nlgst1-1*** **dsRNA concentration in diets with GST activity of** ***nlgst1-1*** **dsRNA-fed BPHs.** BPH nymphs (second to third instar) were reared on the artificial diets with various concentrations of *nlgst1-1* dsRNA for three days, all the survival nymphs of dsRNA-fed BPHs were collected for GST activity assay with a spectrophotometer. The correlation relationship between *nlgst1-1* dsRNA concentration in the diets and GST activity was analyzed and plotted (*p*<0.05). Data used in the analyses were the means from at least three independent experiments.
(TIF)

Figure S7 ***nlgst1-1*** **dsRNA pre-fed BPHs become more sensitive to the diets with gramine.** Gramine was dissolved in artificial diets at the indicated concentrations (GR2 = 25 µg/ml and GR3 = 50 µg/ml). BHPs that had been pre-fed on diets containing 0 (0.8 µg/ml *GFP*-dsRNA, used as control), 0.2 (dsRNA1), 0.4 (dsRNA2) and 0.8 (dsRNA3) µg/ml *nlgst1-1* dsRNA for 3 day were transferred to new diets with the indicated concentrations of gramine and to the normal control diet without dsRNA and gramine. The dsRNA pre-fed BPHs were continually reared on these new diets for an additional 3 days in the controlled growth chambers (90% r.h., 28°C, 14 hr photoperiod). The mortalities of the dsRNA pre-fed BPHs transferred to the new diets were plotted at day 4 (**A**), day 5 (**B**) and day 6 (**C**) post feeding. Data represent the means ± SD from three independent experiments. * and ** indicate significance at *P*<0.05 and *P*<0.01, respectively. “ns” indicates not significant (*P*>0.05).
(TIF)

Figure S8 **Correlations of gramine concentration with the mortality of** ***nlgst1-1*** **dsRNA-fed BPHs at different days post feeding (d.p.f.).** BPH nymphs had been fed on artificial diets containing 0.8 µg/ml *GFP*-dsRNA (0 µg/ml *nlgst1-1* dsRNA, used as control), 0.2 µg/ml (**A**), 0.4 µg/ml (**B**) and 0.8 µg/ml (**C**) *nlgst1-1* dsRNA for three day, the dsRNA pre-fed BPHs were then fed on diets containing 0, 12.5, 25 and 50 µg/ml of gramine for an additional 3 days. BPH mortality was recorded from day 4 after dsRNA feeding and was plotted as a function of the diets containing various concentrations of gramine. Data used in the analyses were the means from at least three independent experiments.
(TIF)

Figure S9 ***nlgst1-1*** **dsRNA decreases the body weight grain of dsRNA pre-fed BPHs.** *nlgst1-1* dsRNA was dissolved in artificial diets at the concentrations of 0 (0.8 µg/ml *GF*P-dsRNA), 0.2, 0.4 and 0.8 µg/ml. BPH nymphs were fed on these liquid diets in the controlled growth chambers (90% r. h., 28°C, 14 h light/10 h dark). Fresh diets containing various concentrations of *nlgst1-1* dsRNA were exchanged every day. After 3 days of feeding, all dsRNA-fed BPHs were weighed as their initial weight. The nymphs were then transferred to rice seedlings (cultivar TN, containing 0.26 mg/g.dw gramine) to be continually reared for an additional 5 days. Subsequently, each nymph was weighed again every day. The increase of body weight of dsRNA pre-fed BPHs was calculated as the following: W_t+1_–W_t_ (W: body weight, t is sampling time when the BPHs were weighted, t = 0, 1, 2, 3, 4 (day). When t = 0, W_0_ is the initial weight of dsRNA pre-fed BPHs before being reared on rice plants in each dsRNA treatment). * indicates significance at *P*<0.05.
(TIF)

Table S1 **Primers used in qRT-PCR assay for other eight glutathione-S-transferase (GST)-related genes in brown planthoppers (BPHs).**
(DOC)
