# Supplementary figures and images for: Glutathione S-Transferase of Brown Planthoppers (Nilaparvata lugens) Is Essential for Their Adaptation to Gramine-Containing Host Plants
Source: PLoS One. 2013 May 20;8(5):e64026. doi: 10.1371/journal.pone.0064026 (PMC3659104; doi:10.1371/journal.pone.0064026)

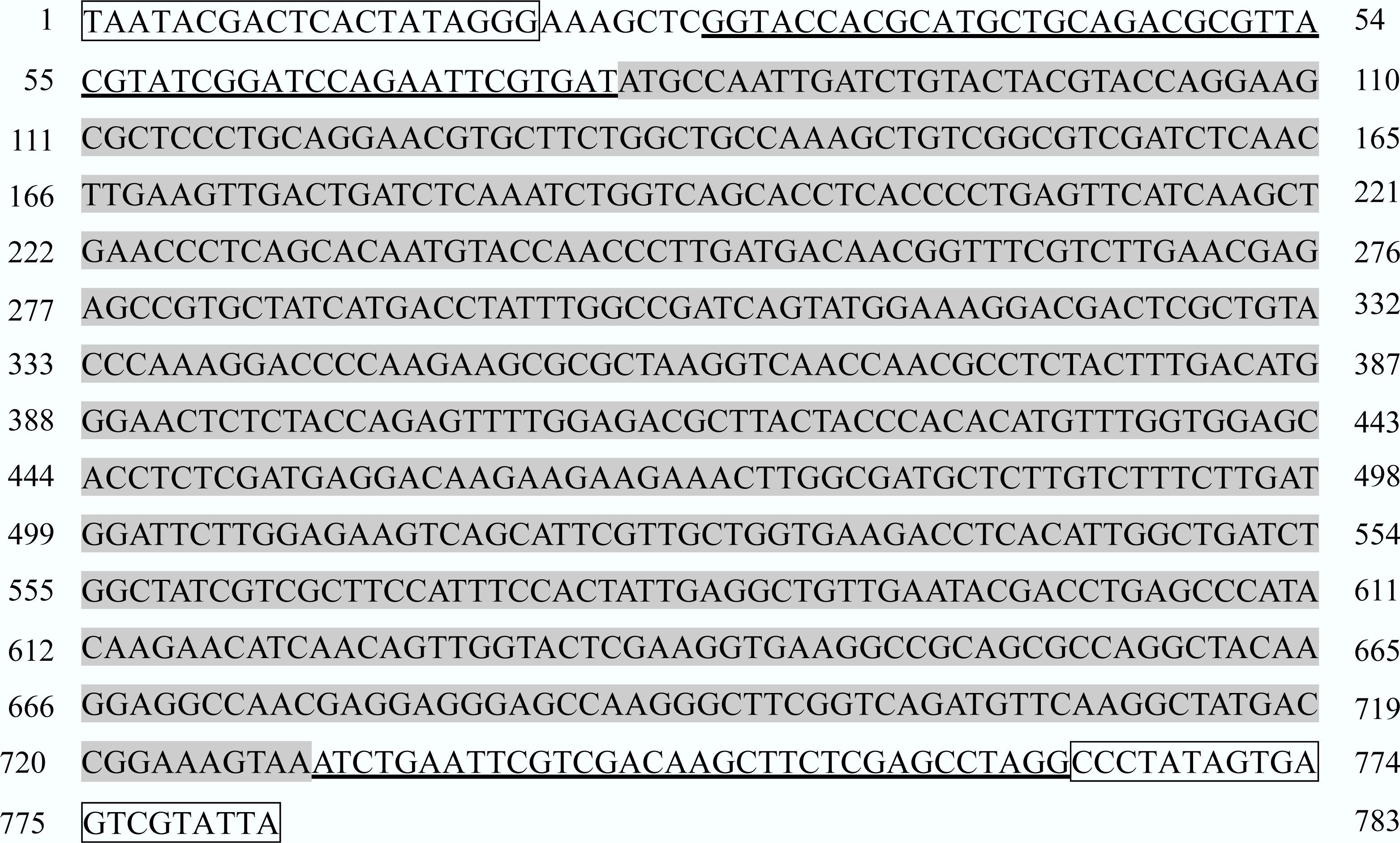

Supplement: Figure S1 — Sequence information of pDrive- nlgst1-1 . T7 RNA polymerase promoter (within frame): bases 1–20 and 764–783; multiple cloning sites (underline): bases 28∼78 and 730∼763; nlgst1-1 ORF region (in gray): bases 79–729. pDrive-nlgst1-1 flanked by T7 polymerase promoter sequences was used as the template for in vitro synthesis of nlgst1-1 dsRNA. Forward and reverse primers used to amplify nlgst1-1 cDNA template were 5′-TAATACGACTCACTATAGGGAAAGCT-3′ (bases 1-26 ) and 5′-TAATACGACTCACTATAGGGCCTAGGCTCGAGAAGCT-3′ (bases 747–783 ), respectively. (TIF) [file pone.0064026.s001.tif]

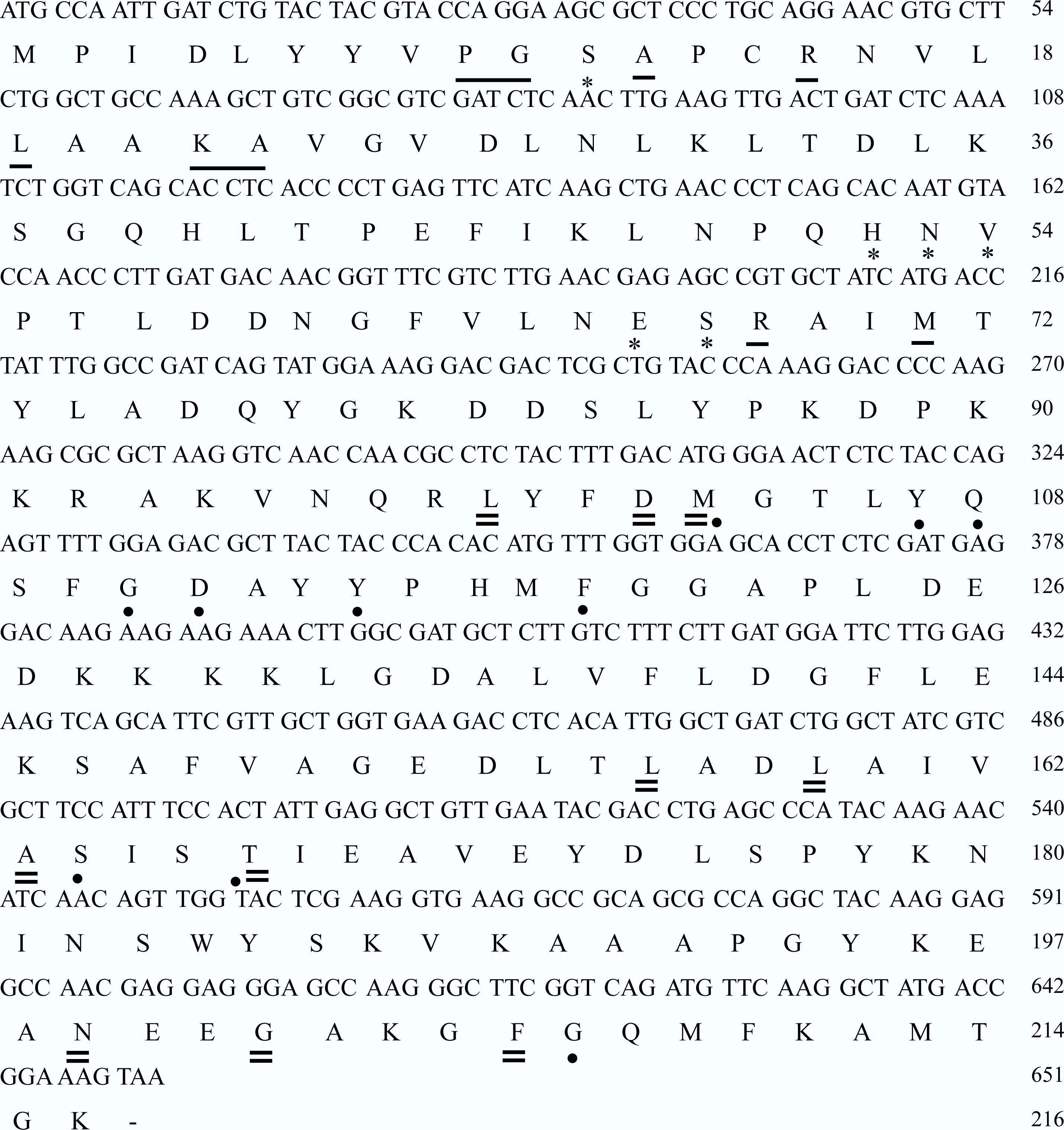

Supplement: Figure S2 — Deduced amino acid sequence of nlgst1-1 protein. Asterisks (below) indicate GSH binding site (G-site) (11, 52.54, 66.67); black dots (below) indicate substrate binding pocket (H-site) (103, 107…108, 111…112, 115,119, 164, 167, 206). The underlined amino acid residues indicate C-terminal domain interface (9.10, 12, 15, 19, 22.23, 68, 71). The double-underlined amino acid residues indicate N-terminal domain interface (99, 102.103, 156, 159, 163, 167, 199, 202, 206). A horizontal dash indicates terminator codon. (TIF) [file pone.0064026.s002.tif]

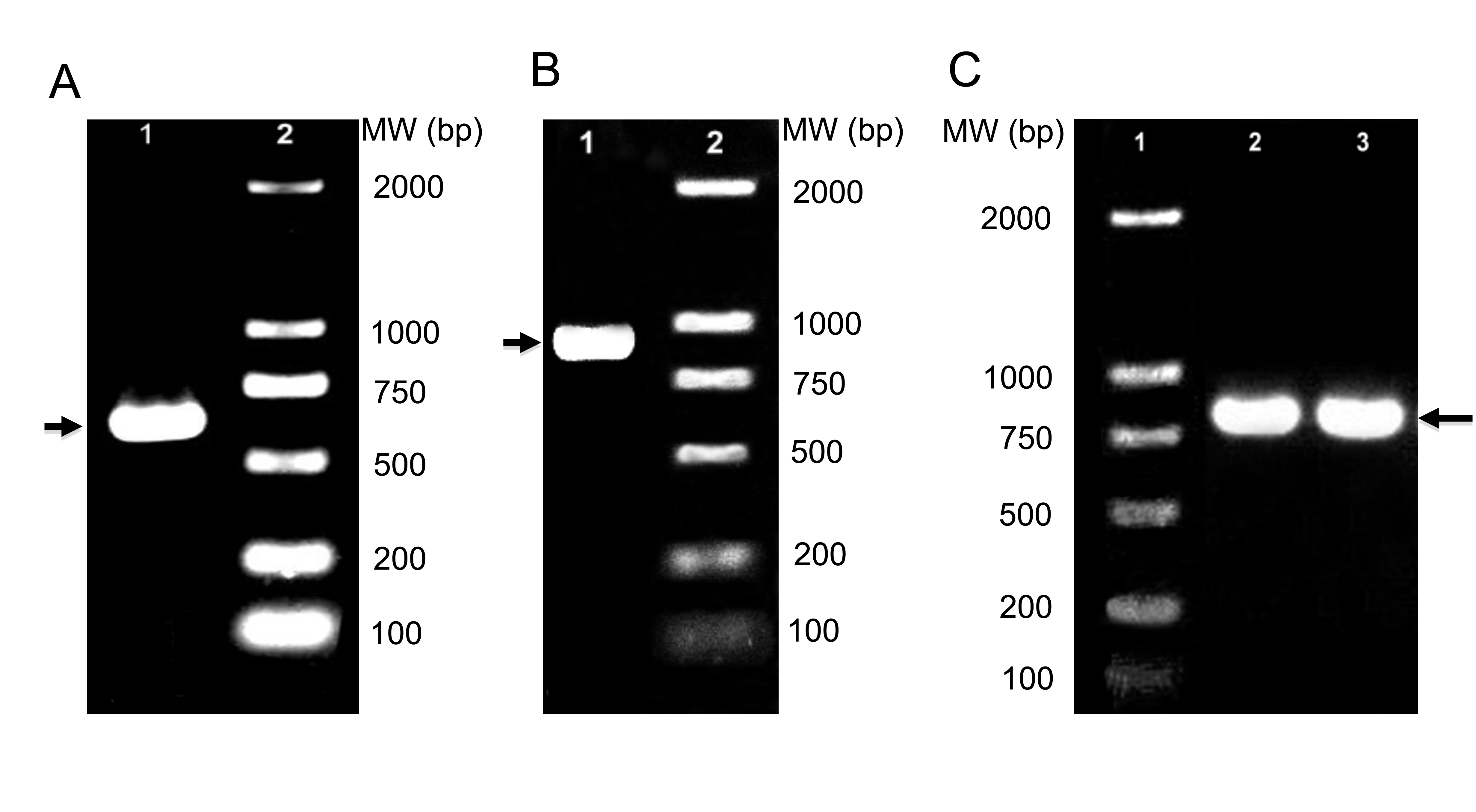

Supplement: Figure S3 — In vitro generation of dsRNA that targets the knockdown of brown planthopper (BPH) glutathione S-transferase (GST) gene nlgst1-1 . Agarose gel (1%) electrophoresis of nlgst1-1 cDNA (PCR amplification product) (A), in vitro transcribed DNA template using pDrive- nlgst1-1 plasmid as templates (B), and in vitro transcribed nlgst1-1 dsRNA (C). The expected bands are indicated in lane 1 in panels (A) and (B), and in lane 2 and lane 3 in panel (C) with arrows. Lane 2 in panels (A) and (B) and Lan1 in panel (C) are DNA molecular weight (MW) markers; the numbers indicate the sizes (bp) of the bands in the markers. (TIF) [file pone.0064026.s003.tif]

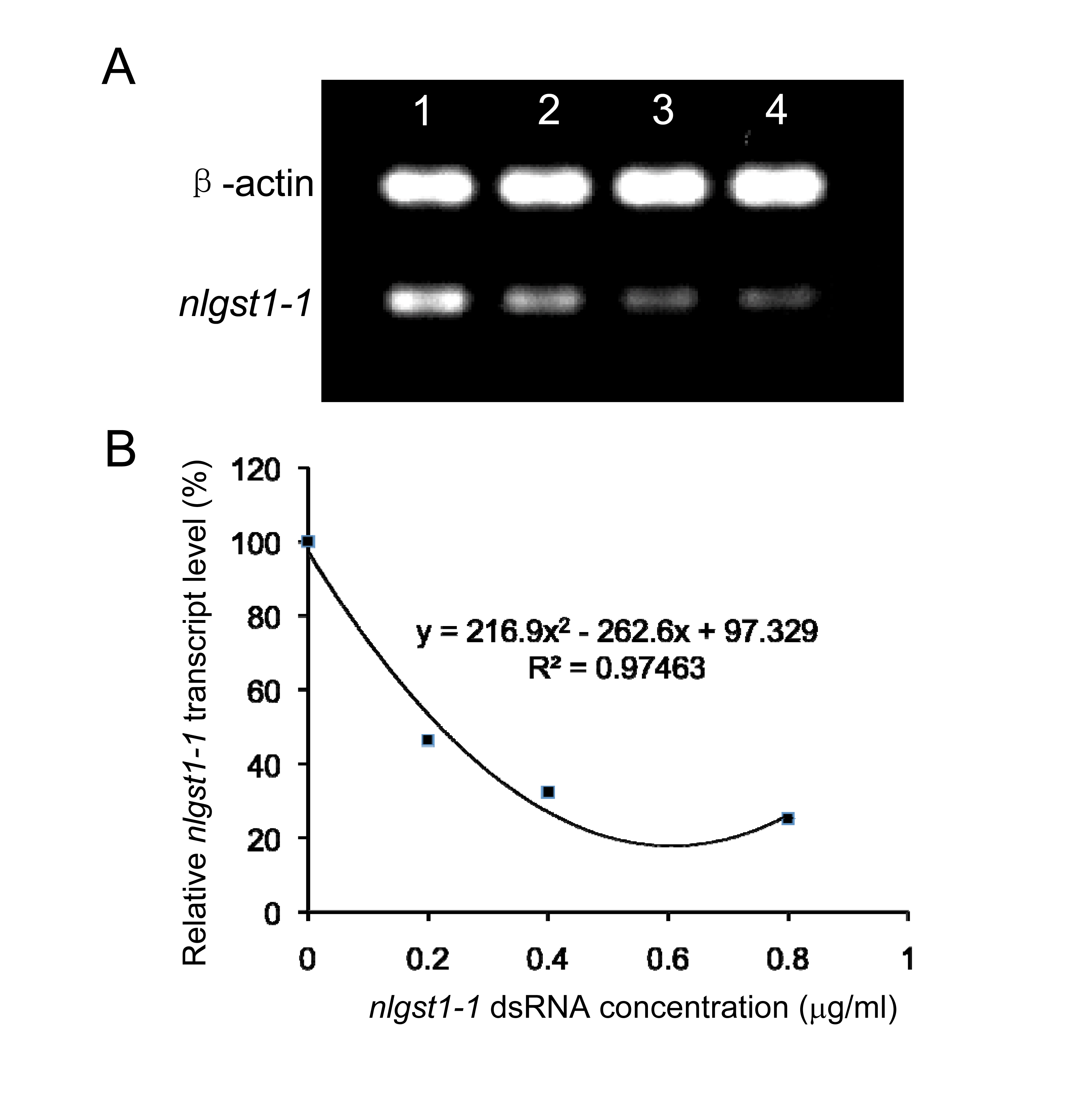

Supplement: Figure S4 — dsRNA-mediated knockdown of nlgst1-1 transcript in BPHs fed with diets containing the indicated concentrations of nlgst1-1 dsRNA. A: Semi-quantitative RT-PCR products (nlgst1-1 transcript) from rice BPH nymphs fed on diets containing various concentrations of the targeted dsRNAs. Lane 1 to lane 4 represents nlgst1-1 transcript from BPH nymphs fed on the diets containing 0 (0.8 µg/ml GFP-dsRNA, used as control), 0.2, 0.4 and 0.8 µg/ml nlgst1-1 dsRNA, respectively. B: correlation of nlgst1-1 transcript level with the concentration of nlgst1-1 dsRNA (P<0.05). (TIF) [file pone.0064026.s004.tif]

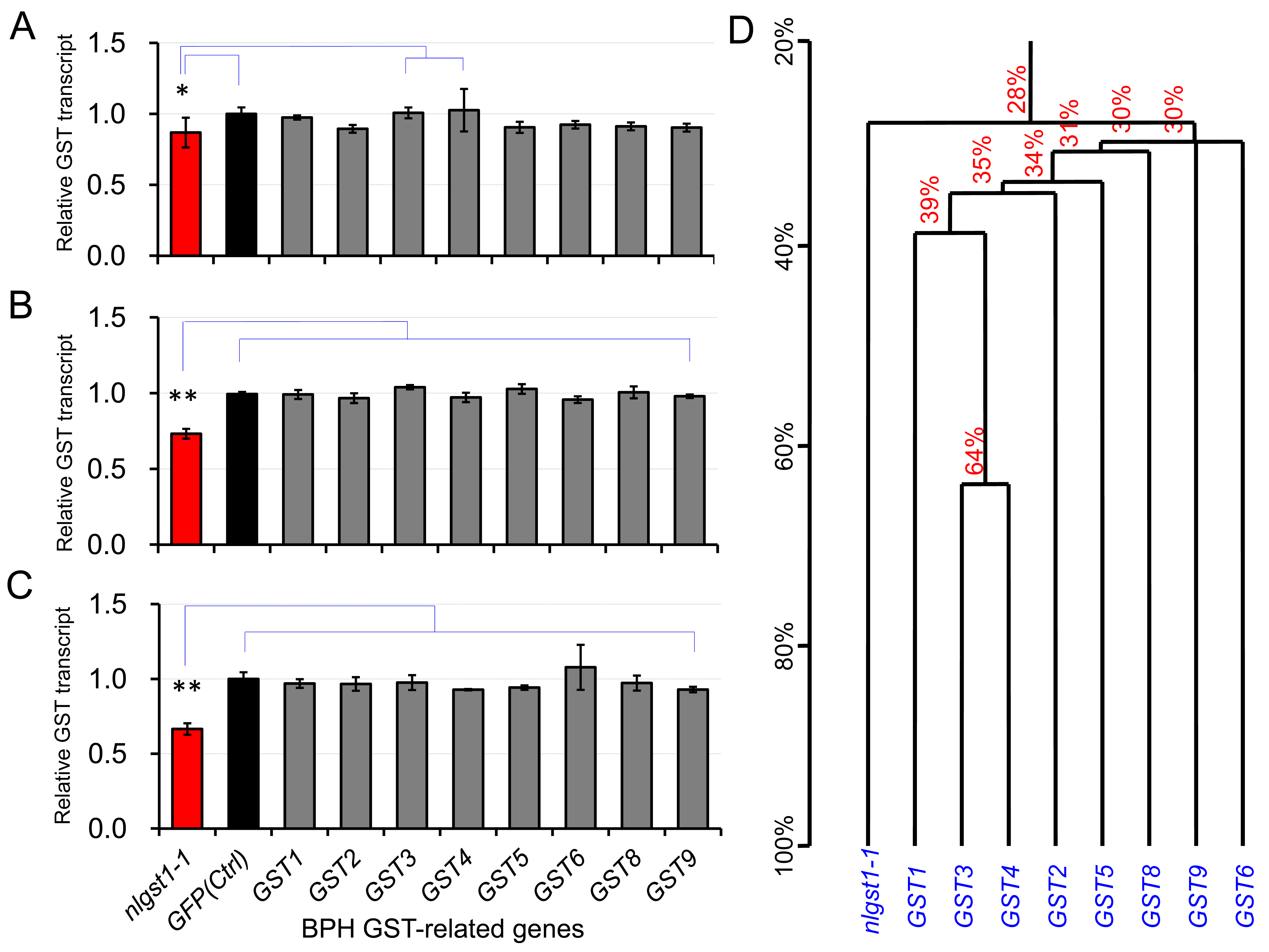

Supplement: Figure S5 — nlgst1-1 dsRNA has no effect on other GST-related genes in rice BPHs. The BPHs were fed with the diets containing 0.2 µg/ml (A), 0.4 µg/ml (B) and 0.8 µg/ml (C) nlgst1-1 and GFP-dsRNAs. The dsRNA-fed PBHs were then used to analyze nlgst1-1 transcript level by qRT-PCR approach as described in the text. The detailed information about GST1 to GST9 was shown in Table S1 and sequence homology (%) among the GST genes was compared with DNAMAN software and shown in panel D. The homology of nlgst1-1 and GST1-GST6, GST8 and GST9 is 9.51%, 21.72%, 32.90%, 16.74%, 34.36%, 20.11%, 33.87% and 34.67%, respectively. The nlgst1-1 transcript level in GFP-dsRNA fed BPHs was used as control [GFP (Ctrl)] and normalized as 1. Data represent the means ± standard deviation from three independent experiments. * and ** indicates significance at P<0.05 and P<0.01, respectively. (TIF) [file pone.0064026.s005.tif]

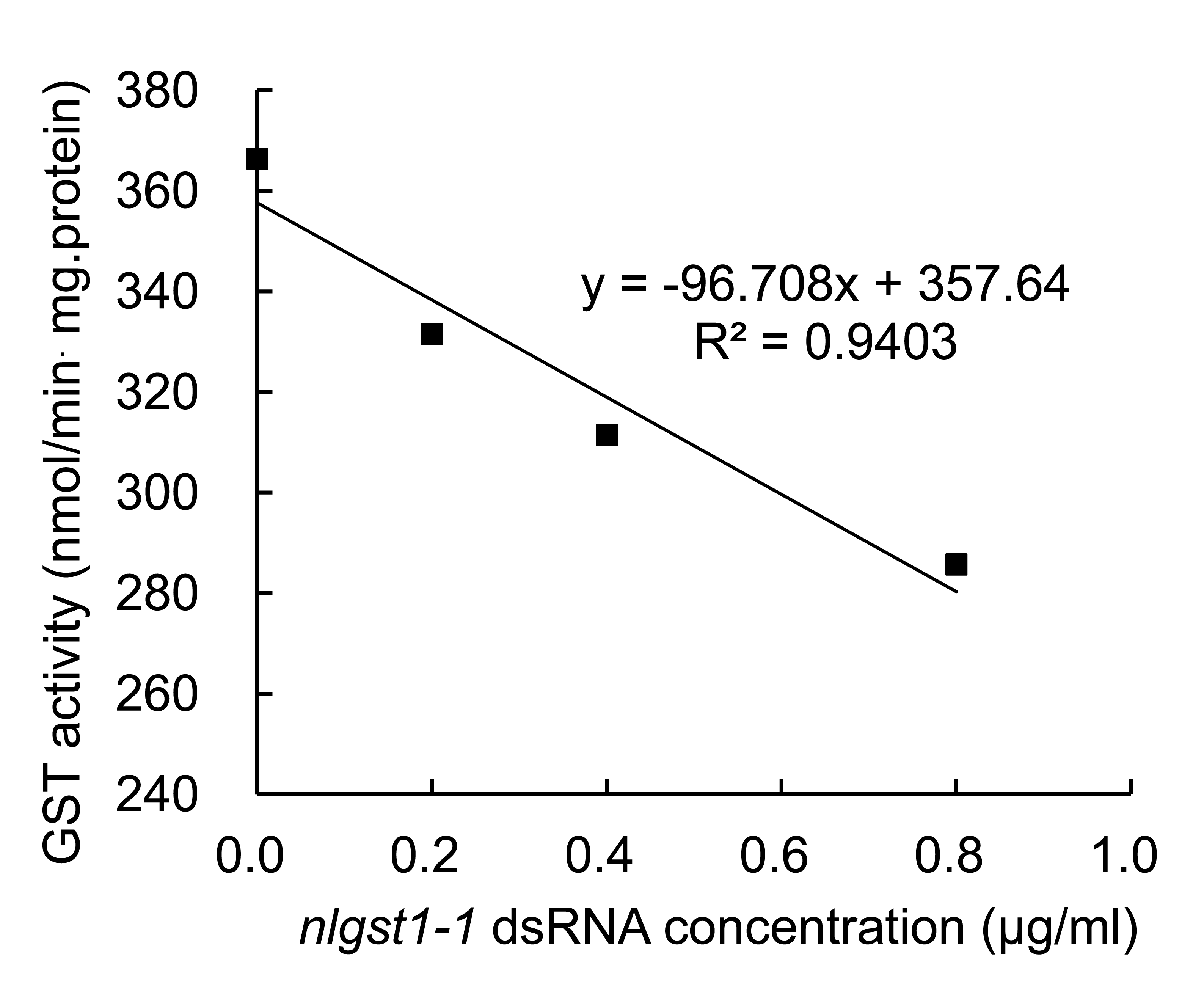

Supplement: Figure S6 — Correlation of nlgst1-1 dsRNA concentration in diets with GST activity of nlgst1-1 dsRNA-fed BPHs. BPH nymphs (second to third instar) were reared on the artificial diets with various concentrations of nlgst1-1 dsRNA for three days, all the survival nymphs of dsRNA-fed BPHs were collected for GST activity assay with a spectrophotometer. The correlation relationship between nlgst1-1 dsRNA concentration in the diets and GST activity was analyzed and plotted (p<0.05). Data used in the analyses were the means from at least three independent experiments. (TIF) [file pone.0064026.s006.tif]

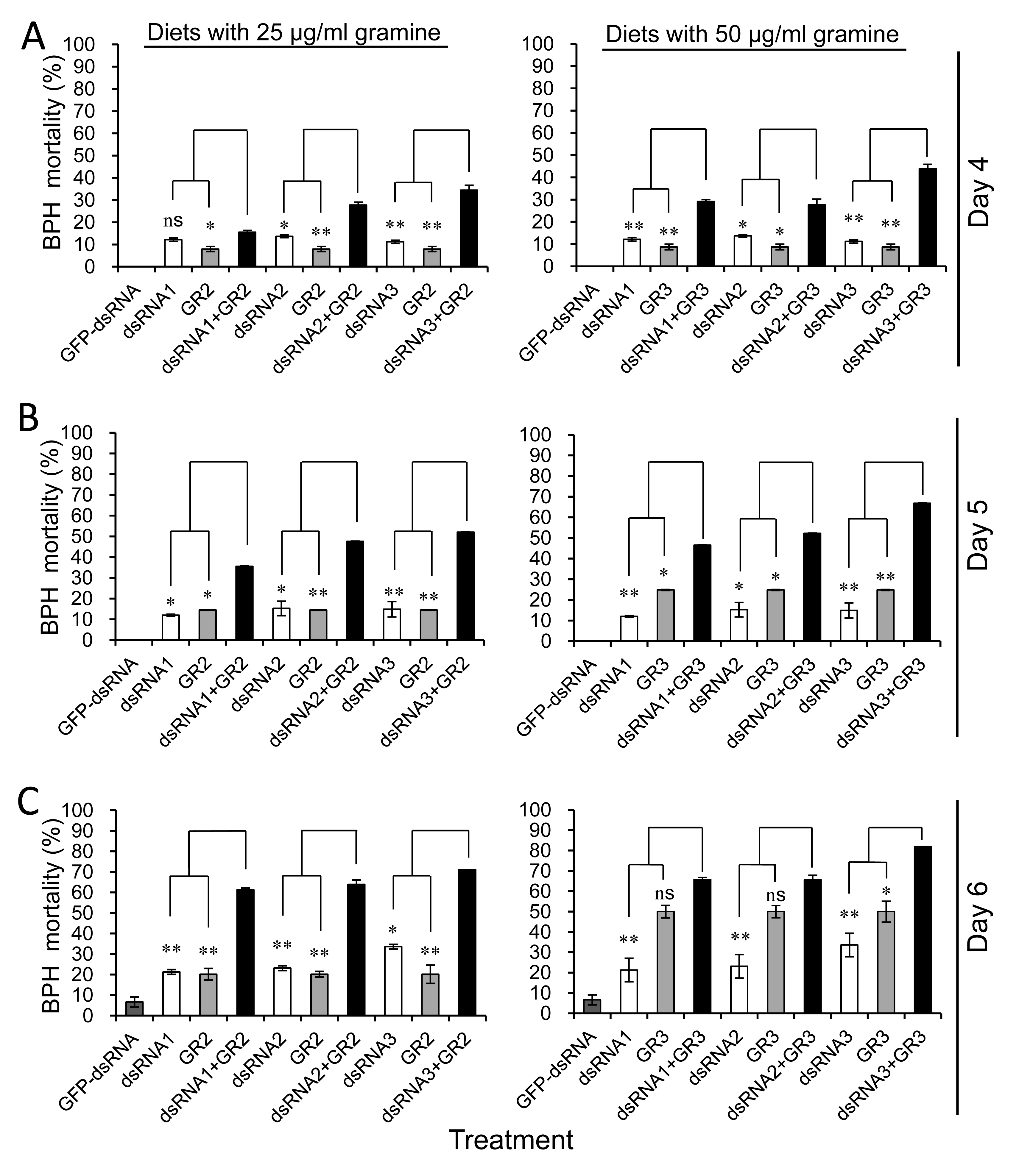

Supplement: Figure S7 — nlgst1-1 dsRNA pre-fed BPHs become more sensitive to the diets with gramine. Gramine was dissolved in artificial diets at the indicated concentrations (GR2 = 25 µg/ml and GR3 = 50 µg/ml). BHPs that had been pre-fed on diets containing 0 (0.8 µg/ml GFP-dsRNA, used as control), 0.2 (dsRNA1), 0.4 (dsRNA2) and 0.8 (dsRNA3) µg/ml nlgst1-1 dsRNA for 3 day were transferred to new diets with the indicated concentrations of gramine and to the normal control diet without dsRNA and gramine. The dsRNA pre-fed BPHs were continually reared on these new diets for an additional 3 days in the controlled growth chambers (90% r.h., 28°C, 14 hr photoperiod). The mortalities of the dsRNA pre-fed BPHs transferred to the new diets were plotted at day 4 (A), day 5 (B) and day 6 (C) post feeding. Data represent the means ± SD from three independent experiments. * and ** indicate significance at P<0.05 and P<0.01, respectively. “ns” indicates not significant (P>0.05). (TIF) [file pone.0064026.s007.tif]

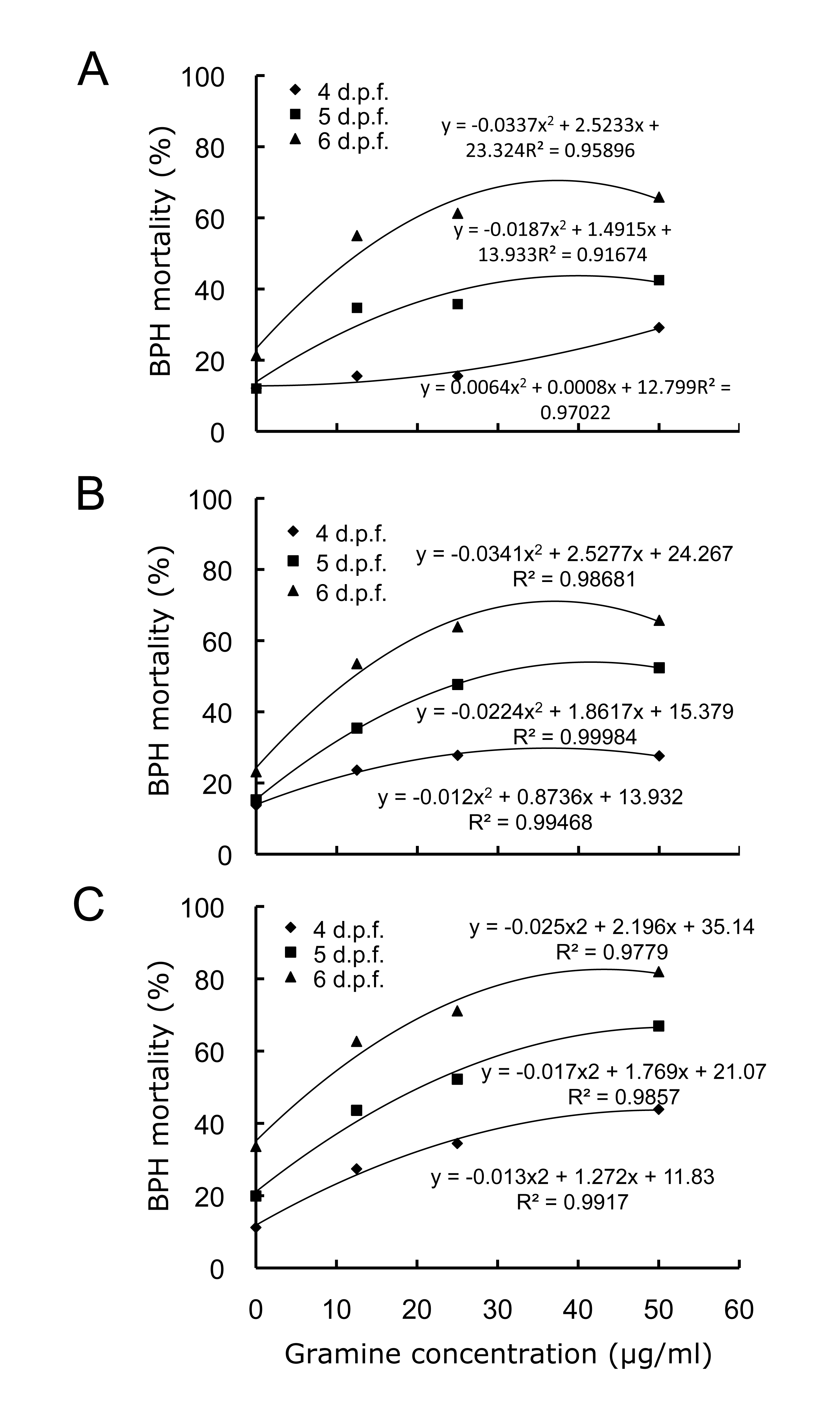

Supplement: Figure S8 — Correlations of gramine concentration with the mortality of nlgst1-1 dsRNA-fed BPHs at different days post feeding (d.p.f.). BPH nymphs had been fed on artificial diets containing 0.8 µg/ml GFP-dsRNA (0 µg/ml nlgst1-1 dsRNA, used as control), 0.2 µg/ml (A), 0.4 µg/ml (B) and 0.8 µg/ml (C) nlgst1-1 dsRNA for three day, the dsRNA pre-fed BPHs were then fed on diets containing 0, 12.5, 25 and 50 µg/ml of gramine for an additional 3 days. BPH mortality was recorded from day 4 after dsRNA feeding and was plotted as a function of the diets containing various concentrations of gramine. Data used in the analyses were the means from at least three independent experiments. (TIF) [file pone.0064026.s008.tif]

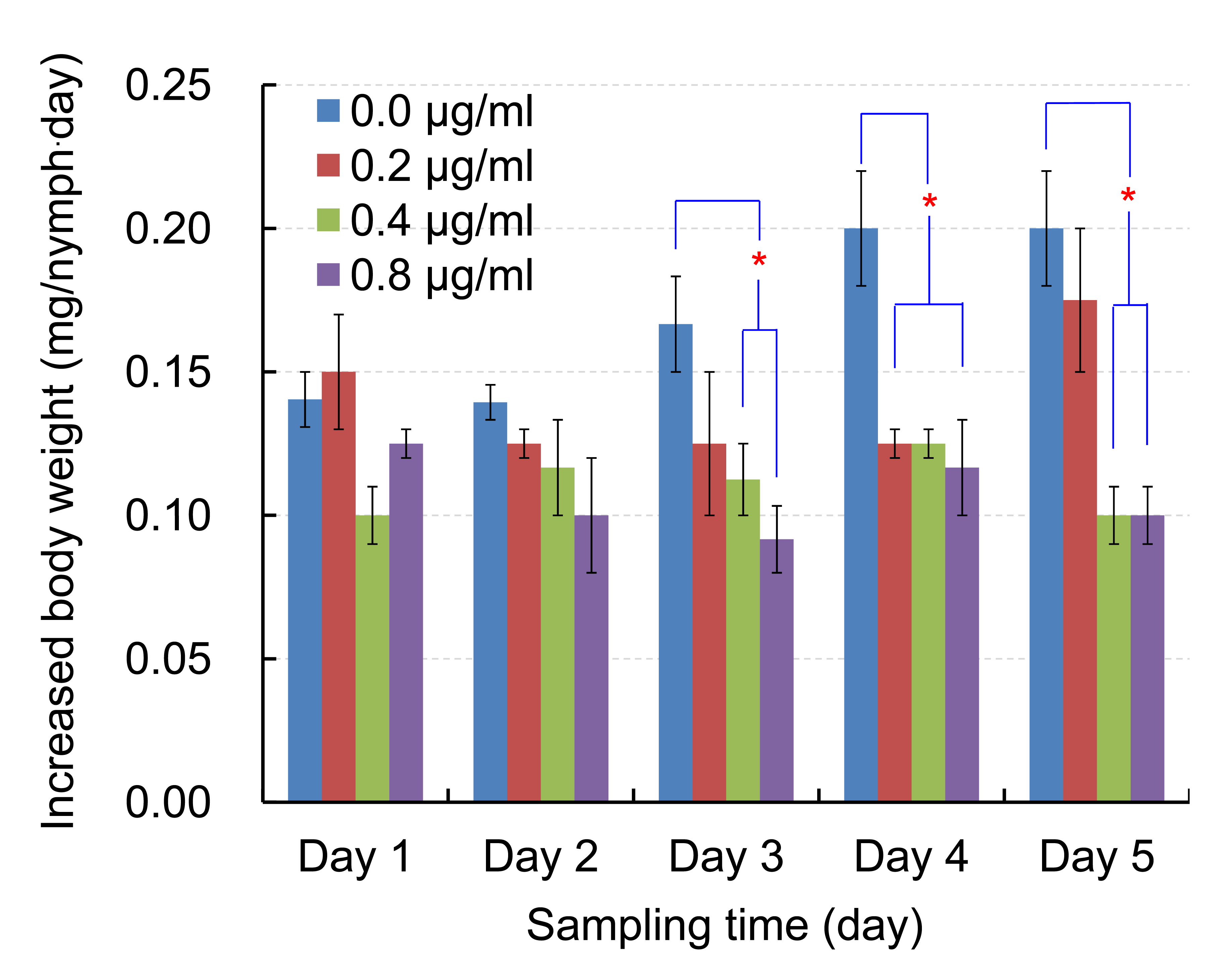

Supplement: Figure S9 — nlgst1-1 dsRNA decreases the body weight grain of dsRNA pre-fed BPHs. nlgst1-1 dsRNA was dissolved in artificial diets at the concentrations of 0 (0.8 µg/ml GFP-dsRNA), 0.2, 0.4 and 0.8 µg/ml. BPH nymphs were fed on these liquid diets in the controlled growth chambers (90% r. h., 28°C, 14 h light/10 h dark). Fresh diets containing various concentrations of nlgst1-1 dsRNA were exchanged every day. After 3 days of feeding, all dsRNA-fed BPHs were weighed as their initial weight. The nymphs were then transferred to rice seedlings (cultivar TN, containing 0.26 mg/g.dw gramine) to be continually reared for an additional 5 days. Subsequently, each nymph was weighed again every day. The increase of body weight of dsRNA pre-fed BPHs was calculated as the following: Wt+1–Wt (W: body weight, t is sampling time when the BPHs were weighted, t = 0, 1, 2, 3, 4 (day). When t = 0, W0 is the initial weight of dsRNA pre-fed BPHs before being reared on rice plants in each dsRNA treatment). * indicates significance at P<0.05. (TIF) [file pone.0064026.s009.tif]
