# Supplementary material for: Glutathione S-Transferase of Brown Planthoppers (Nilaparvata lugens) Is Essential for Their Adaptation to Gramine-Containing Host Plants
Source: PLoS One. 2013 May 20;8(5):e64026. doi: 10.1371/journal.pone.0064026 (PMC3659104; doi:10.1371/journal.pone.0064026)
Supplement: Table S1 — Primers used in qRT-PCR assay for other eight glutathione-S-transferase (GST)-related genes in brown planthoppers (BPHs). (DOC) [file pone.0064026.s010.doc]

Table S1. Primers used in qRT-PCR assay for other eight glutathione-S-transferase (GST)-related genes in rice brown planthoppers (BPHs).

| Gene | ^a^TUG id | Forward | | Reverse |
| --- | --- | --- | --- | --- |
| ^b^*GST1* | FUKV2O402FFGSC | | AAACAAGTACACTACCATGTCCA | TCAAATTGAGGGCTATTCTAACC |
| *GST2* | contig01586 | | CTTCGCCTGTATAGTATGCGTTTC | GCTGGTGTACCATTCTGGCTTCT |
| *GST3* | contig01686 | | GCCTTCAATAAAGCCAACAACTC | TTTCCCATTCATCCCTTCCAGCA |
| *GST4* | contig02241 | | GGGGAGTTGAGCAAGATTGT | GTTCTCCCAAACCAGCAAAG |
| *GST5* | contig02242 | | GCTGGGAGTAATGAGTGGGAGGA | ATTTGCTGATGGATGATCGGAAG |
| *GST6* | contig03174 | | CCTCTTTATTCGTGCTTGGCTCT | CGTGATGTCCACAGGTGCTATTT |
| *GST8* | contig04632 | | TTTGGACATTGAAAGGGTTAGACGA | AACAGGTTGACCGCCAGGAAGTA |
| *GST9* | contig04872 | | GAGCTGTCGGTGTTCCCGTTGAA | TGGCTTTCTGTTGTGGGTTCCTC |

^a^TUG: tentative unique genes [1]. ^b^ GST-related genes *GST1* to *GST6* and *GST8* to *GST9* that may encode the corresponding GST1 to GST6 and GST8 to GST9 in BPHs, respectively. GST1: GST theta, putative GST in *Pediculus humanus corporis*; GST2: predicted GST that is similar to CG6781-PA of *Apis mellifera*; GST3: GST-like protein of *Toxoptera citricida*; GST4: predicted GST that is similar to GST-like protein of *Acyrthosiphon pisum*; GST5: Bla g 5 variant allergen in *Blattella germanica*; GST6: predicted GST that is similar to ganglioside-induced differentiation-associated-protein 1 in *Tribolium castaneum*; *GST7*: corresponds to the identified/named GST gene *nlgst1-1* in *Nilaparvata lugens* (Accession No.: AF448500); GST8: hypothetical protein LOC100163775 in *Acyrthosiphon pisum*; GST9: predicted GST that is similar to GST of *Tribolium castaneum..* The detailed information about GST-related genes *GST1-GST9* in BPHs can be found in the previous report [1].

1. Bass C, Hebsgaard MB, Hughes J (2012) Genomic resources for the brown planthopper, *Nilaparvata lugens*: Transcriptome pyrosequencing and microarray design. Insect Science 19: 1-12.
